# Integrative insights into the role of CAV1 in ketogenic diet and ferroptosis in pancreatic cancer

**DOI:** 10.1038/s41420-025-02421-z

**Published:** 2025-04-04

**Authors:** Xue Liang, Ruofei Tian, Ting Li, Hao Wang, Yifei Qin, Meirui Qian, Jing Fan, Dan Wang, Hong-Yong Cui, Jianli Jiang

**Affiliations:** 1 Department of Cell Biology, National Translational Science Center for Molecular Medicine, Fourth Military Medical University, Xi’an, Shaanxi 710032 China; 2 State Key Laboratory of New Targets Discovery and Drug Development for Major Diseases, Xi’an, Shaanxi 710032 China; 3 State Key Laboratory of Holistic Integrative Management of Gastrointestinal Cancers, Xi’an, Shaanxi 710032 China; 4 Cardiovascular Surgery Department, Shaanxi Provincial People’s Hospital, Xi’an, Shaanxi 710068 China; 5 Institutes of Biomedicine and Department of Cell Biology, Jinan University, Guangzhou, 510632 China; 6 Air Force Hospital of the Northern Theater Command of the People’s Liberation Army of China, Shenyang, Liaoning 110003 China

**Keywords:** Gastrointestinal cancer, Cell death

## Abstract

Pancreatic cancer exhibits high mortality rates with limited therapeutic options. Emerging evidence suggests that the ketogenic diet may act as adjuvant therapy by triggering ferroptosis in cancer cells, though the underlying molecular mechanisms remain unclear. This study aims to investigate the molecular mechanisms linking ketogenic metabolism and ferroptosis, with an emphasis on key regulatory proteins. We demonstrated that pancreatic adenocarcinoma (PAAD) tissues significantly enhanced ketogenic and ferroptosis phenotypes compared to normal tissues, both correlating with poorer patient prognosis. These phenotypes showed strong interdependence mediated by CAV1. In the pancreatic tumor microenvironment, CAV1 was predominantly expressed in tumor cells. Through in vitro cell experiments, we clarified that Na-OHB downregulated CAV1 expression in pancreatic cancer cells, inhibiting the transcription of the CAV1/AMPK/NRF2 downstream ferroptosis-protective genes SLC7A11 and SLC40A1. Additionally, we demonstrated the interaction between CAV1 and SLC7A11 molecules; when CAV1 was downregulated, it affected the stability of SLC7A11, leading to the ubiquitination and degradation of the translated SLC7A11 protein. Through these dual mechanisms, Na-OHB caused Fe^2+^ overload, lipid peroxidation accumulation, and oxidative stress in pancreatic cancer cells, ultimately triggering ferroptosis. In ketogenic diet-fed tumor-bearing mouse models, we also observed a significant increase in lipid peroxidation and other related biomarkers, while CAV1 and SLC7A11 levels were markedly decreased compared to the normal diet group. Our findings identify CAV1 as a pivotal molecular link between ketogenic metabolism and ferroptosis in pancreatic cancer. The multi-level regulatory axis involving CAV1-mediated transcriptional regulation and post-translational modifications provides mechanistic insights into ketogenic diet-induced ferroptosis, suggesting potential therapeutic targets for pancreatic cancer adjuvant treatment.

## Introduction

Pancreatic cancer accounts for almost as many deaths as cases and ranks as the seventh leading cause of cancer-related death [1]. It is anticipated that by 2030, it will emerge as the second most prominent contributor to cancer-related fatalities in the United States [2]. Pancreatic ductal adenocarcinoma accounts for ~90% of pancreatic cancer [3]. Chemotherapy and surgery are the primary therapeutic options for pancreatic cancer [4]. However, only 15–20% of patients are eligible for surgery at the time of diagnosis [5]. Therapeutic approaches targeting metabolic heterogeneity have sparked increasing interest [6, 7], and the metabolic environment within pancreatic cancer is dysregulated [8]. From this perspective, elucidating the molecular mechanisms underlying these emerging adjunctive therapeutic approaches is imperative for the treatment of pancreatic ductal adenocarcinoma.

Ferroptosis, a distinctive form of non-apoptotic cell death, is characterized by a reliance on Fe^2+^ and the consequent peroxidation of lipids [9, 10]. This form of cell death can be triggered by a variety of agents [11]. The metabolism of fatty acids also plays a crucial role in the initiation and propagation of ferroptosis [12]. Ferroptosis serves as a significant tumor suppressor [13, 14], although tumors have evolved mechanisms to evade it. Additionally, ferroptosis mediates the tumor-suppressive effects of several conventional cancer therapies [15]. Thus, the induction of ferroptosis may be a promising therapeutic strategy for eliminating cancers with specific characteristics [16].

A ketogenic diet is characterized as a diet containing 80–90% fat but <5% carbohydrate, resulting in elevated levels of ketone bodies in the bloodstream. Ketone bodies include acetoacetate, β-hydroxybutyrate, and acetone, primarily transported in the blood as β-hydroxybutyrate [17]. The ketogenic diet has been reported to enhance the efficacy of various antitumor therapies with mechanisms attributed to restricted glucose supply to tumor cells, modulation of signaling pathways, or an overall reduction in inflammation [18]. In addition, in a cachectic mouse model of cancer, the ketogenic diet increased lipid peroxidation, leading to a systemic redox imbalance, while saturation of the glutathione pathway within tumor cells induced ferroptosis [19].

In this study, we systematically collected ketogenic metabolism-related genes from the gene set enrichment analysis (GSEA) database and ferroptosis-related genes from the FerrDb database. Using the Cancer Genome Atlas (TCGA) database data for pancreatic adenocarcinoma (PAAD), we employed various methodologies, including the identification of differentially expressed genes (DEGs), weighted gene co-expression network analysis (WGCNA), univariate Cox regression, the Kaplan–Meier curves, and machine learning, to filter feature-related genes and construct risk models. Receiver operating characteristic (ROC) curves were used to characterize the predictive performance of the models. Next, the single sample gene set enrichment analysis (ssGSEA) algorithm was used to assign phenotypic scores to each sample. We assessed the association between the two phenotypes and identified a key gene, CAV1, by linking them through functional enrichment, correlation analysis, immune infiltration, and protein-protein interaction (PPI) analysis. Single-cell RNA-sequencing (scRNA-seq) data were analyzed to determine the localization of the two phenotypes and risk genes at the cellular level. Multiplex immunofluorescence staining validated the expression of CAV1 on tumor cells. By establishing an extracellular ketogenic culture environment and ketogenic diet-fed mice models, we validated the association between these two phenotypes and their underlying molecular connections. In conclusion, our study elucidates the molecular mechanisms underlying ketogenic metabolism-related ferroptosis in pancreatic cancer and identifies potential targets for treatment.

## Results

### Ketone-stage DEGs and ferroptosis-stage DEGs exhibit similar characteristics

**Fig. 1:**
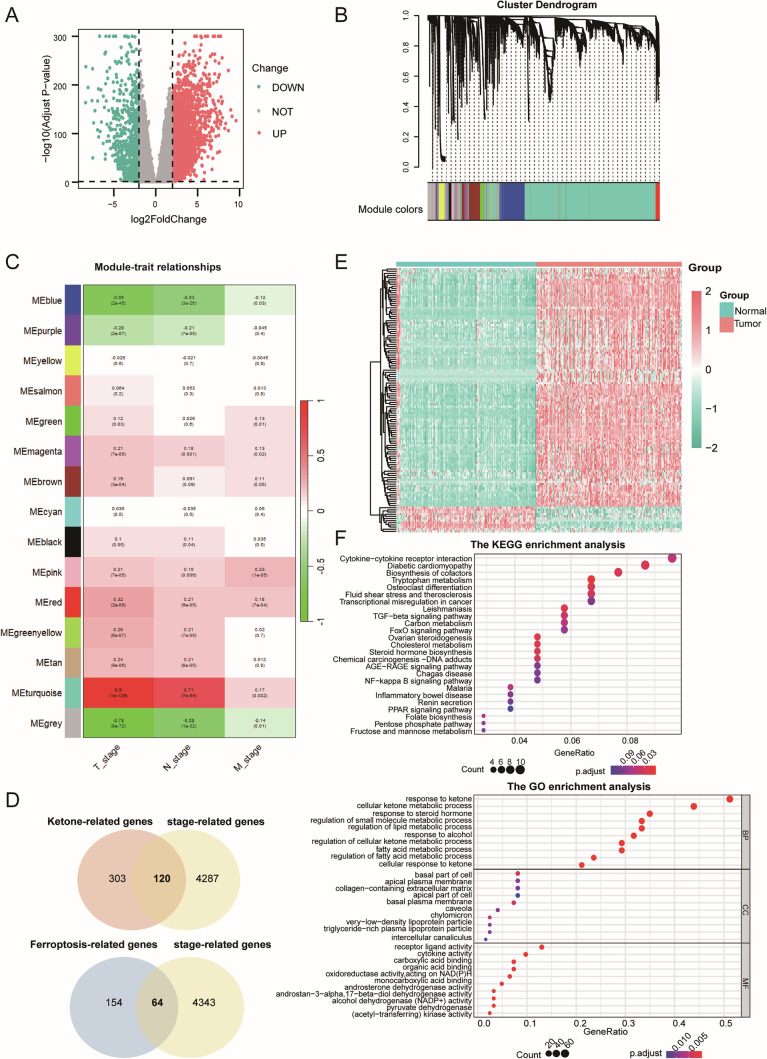
Ketone-stage DEGs and ferroptosis-stage DEGs exhibit similar characteristics. **A** DEGs between PAAD and normal tissues. **B** Hierarchical clustering of genes based on co-expression network. **C** Module-trait relationship of co-expression modules and pathological grades. **D** Identification of ketone-stage DEGs and ferroptosis-stage DEGs. **E** Heatmap of ketone-/ferroptosis-stage DEGs between PAAD and normal tissue. **F** KEGG enrichment (upper) and GO enrichment (lower) of ketone-stage DEGs.

After merging the datasets, we identified 7184 DEGs between PAAD and normal pancreatic tissues, comprising 4831 upregulated and 2353 downregulated genes (Fig. 1A). Based on the sample clustering diagram (Supplementary Fig. 2) and soft threshold (Supplementary Fig. 3A), WGCNA revealed 14 distinct gene expression modules (Fig. 1B), among which the MEturquoise module exhibited strong correlations with T and N stage, with correlation coefficients of 0.9 and 0.71 respectively (Fig. 1C). The 4407 genes within the MEturquoise module were identified as stage-related genes. To further investigate the connection between stage-related genes, ketone body metabolism, and ferroptosis. 120 ketone-stage DEGs were intersected by stage-related genes with ketone-related genes and 64 ferroptosis-stage DEGs in the same way (Fig. 1D). Analysis of these genes between PAAD and normal tissues revealed significant differences (Fig. 1E). GO and KEGG enrichment analyses showed that, in addition to the enrichment of ketone-stage DEGs in pathways associated with ketone metabolism and ferroptosis-stage DEGs in oxidative stress pathways, they shared enrichment in lipid metabolism-related biological processes, caveola cellular components, molecular function of oxidoreductase activity, and some signaling pathways, such as the AGE-RAGE and PPAR signaling pathways (Fig. 1F and Supplementary Fig. 3B). These indicated a certain degree of cross-functionality between ketone-stage DEGs and ferroptosis-stage DEGs, underscoring their intricate interplay.

### Ketone metabolism and ferroptosis genes are reliable indicators for predicting the prognosis of pancreatic cancer patients

**Fig. 2:**
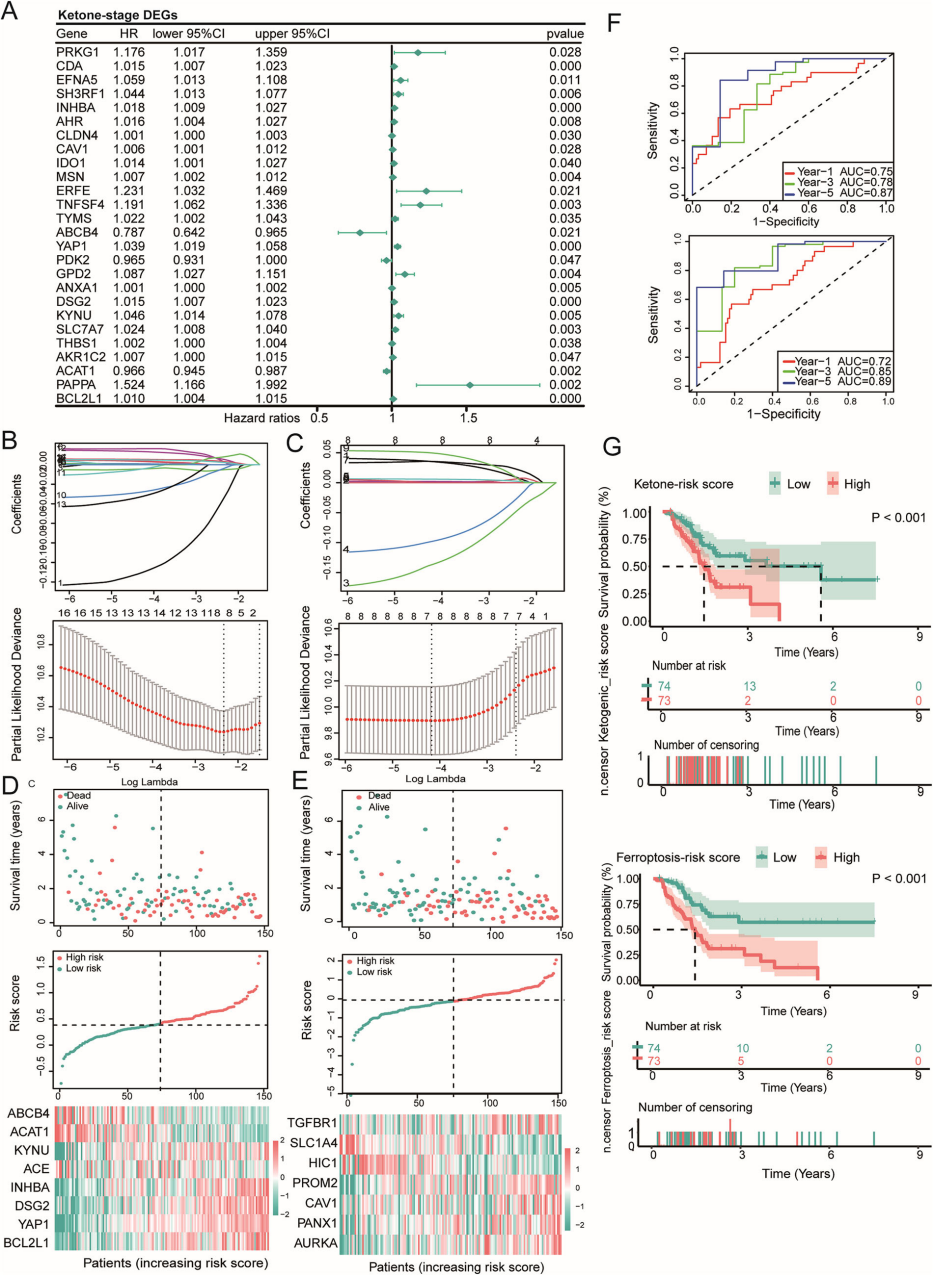
Ketone metabolism and ferroptosis genes are reliable indicators for predicting the prognosis of pancreatic cancer patients. **A** Forest plot of univariate Cox regression of ketone-stage DEGs. **B**, **C** The optimal lambda (upper) and the partial likelihood deviation curve (lower) for the ketogenic risk model (**B**) and the ferroptosis risk model (**C**). **D**, **E** Distribution of the risk score, survival status, and expression profiles of risk genes for the ketogenic risk model (**D**) and the ferroptosis risk model (**E**). **F** Time-dependent ROC curves at 1, 3, and 5 years for the ketogenic risk model (upper) and ferroptosis risk model (lower). **G** Kaplan–Meier curves of the high and low risk groups.

Univariate Cox regression and the Kaplan–Meier method were employed to screen 17 ketone-stage-survival DEGs and nine ferroptosis-stage-survival DEGs (Fig. 2A and Supplementary Fig. 4A–C) that were associated with the prognosis of PAAD patients (*P* value <0.1). LASSO regression further identified eight ketone-risk DEGs, namely, ABCB4, ACAT1, KYNU, ACE, INHBA, DSG2, YAP1, and BCL2L1 (Fig. 2B), and seven ferroptosis-risk DEGs, including TGFBR1, SLC1A4, HIC1, PROM2, CAV1, PANX1, and AURKA (Fig. 2C). These genes were used to construct two prognostic models with risk scores: ketogenic = (0.0023 × BCL2L1) + (0.0081 × YAP1) - (0.0335 × ABCB4) - (0.0051 × ACAT1) + (0.0028 × KYNU) - (0.0037 × ACE) + (0.0053 × INHBA) + (0.0032 × DSG2); ferroptosis = (0.0352 × TGFBR1) - (0.1363 × SLC1A4) - (0.0977 × HIC1) + (0.0059 × PROM2) + (0.0020 × CAV1) + (0.0354 × PANX1) + (0.0495 × AURKA). The distributions of risk scores, survival time, survival status, and expression patterns of the risk genes are displayed (Fig. 2D, E). We evaluated the predictive accuracy of the risk models using ROC curves. The area under the ROC curve (AUC) for ketone risk at 1, 3, and 5 years was 0.75, 0.78, and 0.87, respectively. The AUC for ferroptosis risk at 1, 3, and 5 years was 0.72, 0.85, and 0.89, respectively (Fig. 2F). Kaplan–Meier survival analysis consistently revealed that patients with low-risk scores demonstrated significantly superior outcomes across both risk models (Fig. 2G). These results highlight the risk genes significantly associated with patient’s survival, reflecting their strong predictive capability for patients’ prognosis.

### Ketone metabolism and ferroptosis phenotypes are intrinsically correlated

**Fig. 3:**
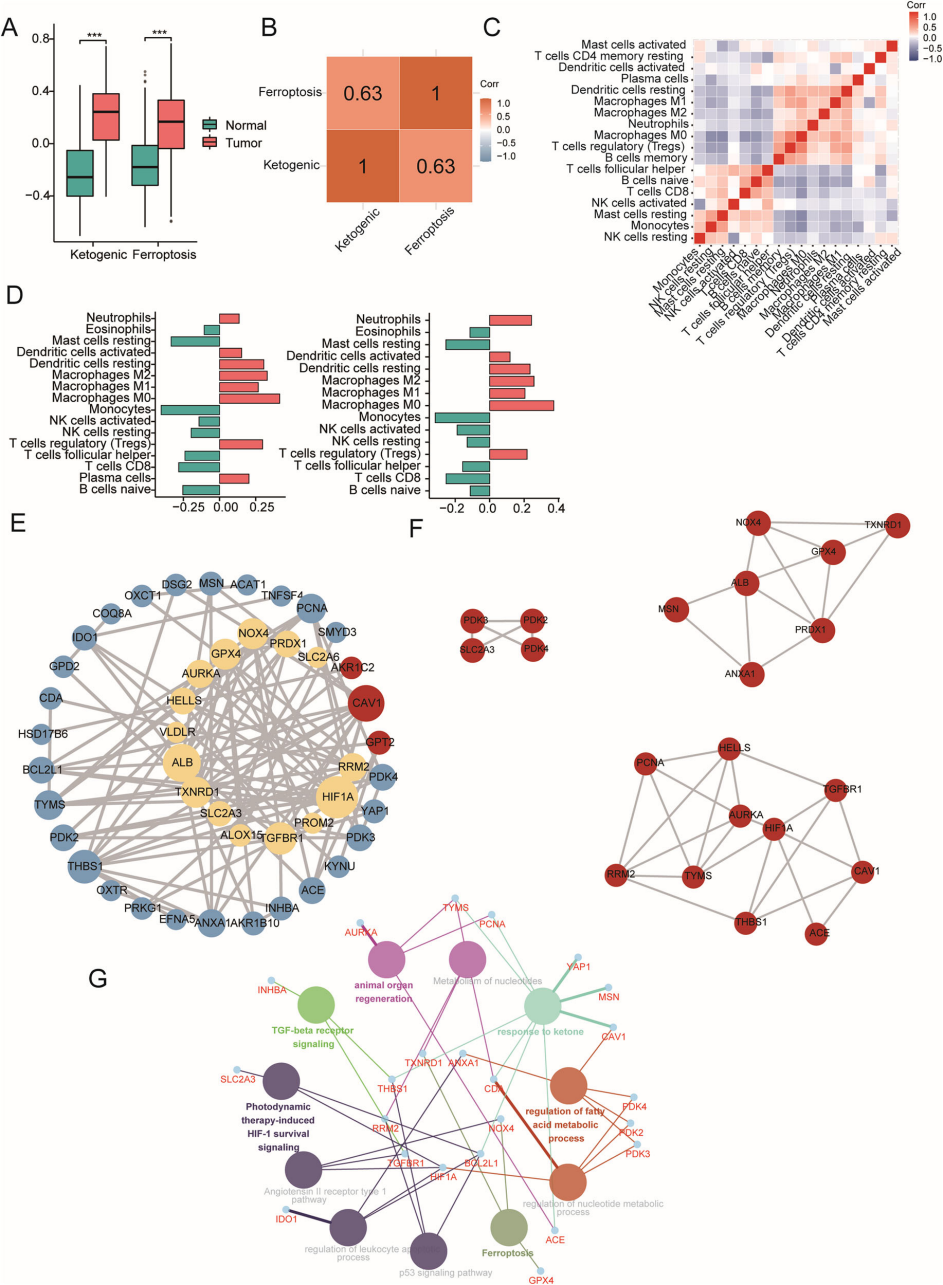
Ketone metabolism and ferroptosis phenotypes are intrinsically correlated. **A** Phenotype scores of PAAD and normal tissues for ketogenic and ferroptosis risk genes. **B** Correlation heatmap of two phenotype scores. **C** Correlation heatmap of 17 infiltrating immune cells. **D** Correlation between two phenotypes and infiltrating immune cells (left: ketogenic phenotype; right: ferroptosis phenotype). **E** PPI network of survival-related genes screened by univariate Cox regression. Ketone-related genes are in yellow, ferroptosis-related genes are in blue, and three shared genes are in red. The size of the bubbles represents the connective degree. Lines between bubbles represent interaction. **F** Three subnetworks with strong interactions, with clustering scores of 3.333 (left upper), 4.333 (right upper), and 4.889 (right lower). **G** GO enrichment of the most clustered subnetwork with its first connective nodes. Pathways are distinguished by colors. ***P < 0.001.

Two phenotypic scores, ketogenic and ferroptosis, were computed for PAAD samples, and they were consistently higher than those for normal tissues (Fig. 3A). A notable positive correlation (correlation coefficient of 0.63) was observed between the two phenotypes (Fig. 3B). The tumor immune microenvironment plays a crucial role in the initiation and progression of tumors [20]. Among 22 types of immune cells, 17 immune cells showed significant differences between PAAD and normal tissues (Supplementary Fig. 5A,B). The infiltration levels of these immune cells manifested in two main correlated modules: one dominated by NK cells, B cells, and activated T cells, and the other by macrophages, DCs, neutrophils, and Tregs (Fig. 3C). The correlation analysis between the phenotypes and infiltrated immune cells demonstrated a consistently positive correlation with the infiltration of macrophages, DCs, neutrophils, and Tregs (Fig. 3D). These findings collectively underscore the close association between ketogenic metabolism, ferroptosis, and the immune microenvironment of pancreatic cancer. To explore the molecular links between ketogenic metabolism and ferroptosis in pancreatic cancer, a PPI network was constructed using the genes identified by univariate Cox regression. This network consisted of 15 ketone-related genes, 27 ferroptosis-related genes, and three common genes, namely, CAV1, AKR1C2, and GPT2 (Fig. 3E). Further calculations identified three subnetworks with strong interactions, with clustering scores of 3.333, 4.333, and 4.889 (Fig. 3F and Supplementary Fig. 5C). The subnetwork that included CAV1, rather than AKR1C2 or GPT2, had the highest clustering score, indicating the most robust interactions within this subnetwork. GO enrichment analysis of this network demonstrated that ketone metabolism and ferroptosis were interconnected through lipid metabolism and TGF-β pathways, in which CAV1 plays a central role (Fig. 3G).

### Single-cell-level expression of the two phenotypes

**Fig. 4:**
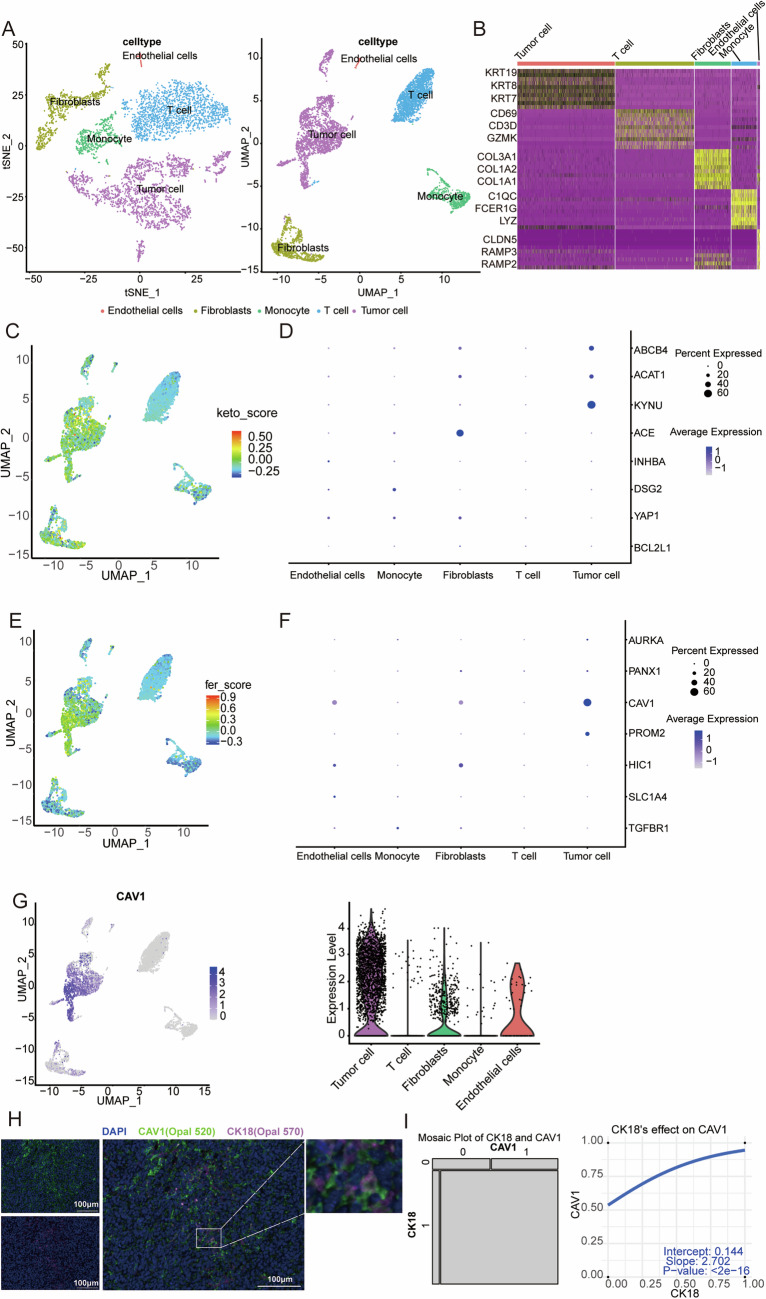
Single-cell-level expression of the two phenotypes. **A** Cell clustering by tSNE and UMAP. **B** Marker genes of each cell type. **C**, **D** Expression of ketogenic phenotype (**C**) and genes (**D**) among cell types. **E**, **F** Expression of ferroptosis phenotype (**E**) and genes (**F**) among cell types. **G** CAV1 expression among cell types. **H** Multiplex immunofluorescence staining of subcutaneously implanted Pan02 cell line tumors. **I** The mosaic plot of positivity rates of CK18 and CAV1 (left) and the logistic bivariate regression curve between CK18 and CAV1 (right).

To explore the heterogeneity of ketogenic and ferroptosis phenotypes at the single-cell level, we reanalyzed published single-cell RNA-seq data on pancreatic cancer. Dimensionality reduction clustering analysis identified five major cell clusters (Fig. 4A), and cell types were categorized based on the marker genes: tumor cells, T cells, endothelial cells, fibroblasts, and monocytes (Fig. 4B). The ketogenic phenotype was primarily observed in tumor cells and fibroblasts (Fig. 4C and Supplementary Fig. 6A). However, individual gene expression varies among different cell types. Notably, three specific genes were predominantly expressed in tumor cells (Fig. 4D). The ferroptosis phenotype was also predominantly expressed in tumor cells (Fig. 4E and Supplementary Fig. 6B). Notably, CAV1 was highly enriched in tumor cells, and its expression may significantly influence the cell type-specific predisposition to the ferroptosis phenotype (Fig. 4F, G). Therefore, through multiplex immunofluorescence staining of tumor tissue sections derived from subcutaneously implanted Pan02 tumor cells in mice, we determined that CAV1 is highly enriched in CK18^+^ epithelial cells (Fig. 4H). Bivariate regression analysis further established a positive causal relationship between CK18 and CAV1 expression, indicating that CK18^+^ epithelial cells have a significantly higher probability of expressing CAV1 compared to CK18^-^ cells (Fig. 4I).

### β-hydroxybutyrate induces ferroptosis of pancreatic cancer cells by downregulating CAV1

**Fig. 5:**
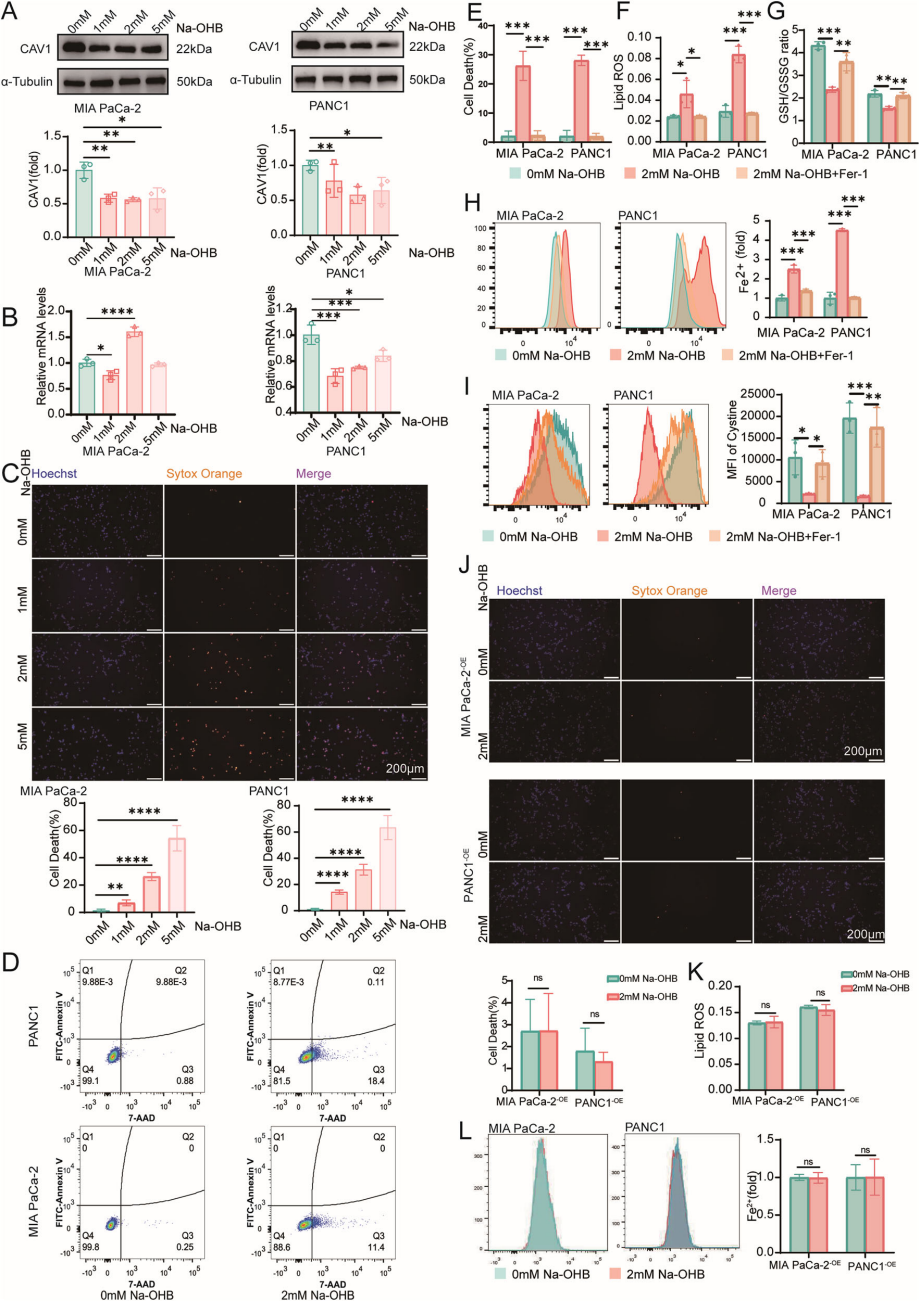
β-hydroxybutyrate induces ferroptosis of pancreatic cancer cells by downregulating CAV1. **A** Western blotting of CAV1 and α-Tubulin after treatment by a gradient concentration (0, 1, 2, and 5 mM) of Na-OHB. **B** qPCR of CAV1 after treatment by a gradient concentration (0, 1, 2, and 5 mM) of Na-OHB. **C** MIA PaCa-2 cells were treated with a gradient concentration of Na-OHB, and dead cells were stained with SYTOX Orange. The bar graph (left) shows the cell death rate of MIA PaCa-2 cells following Na-OHB treatment, while the bar graph (right) presents the cell death rate of PANC1 cells after Na-OHB treatment. **D** Apoptosis and dead cells were detected using flow cytometry. **E** Cells were treated with Na-OHB with or without Fer-1, and dead cells were stained with SYTOX Orange. **F** Lipid ROS levels within cells were measured by flow cytometry. **G** GSH/GSSG ratio of MIA PaCa-2 and PANC1 cells after being treated with Na-OHB/Fer-1. **H** Intracellular Fe^2+^ levels were assessed using flow cytometry. **I** Intracellular cystine was assessed using flow cytometry. **J** CAV1 overexpression cells were treated with 0 mM or 2 mM Na-OHB, and dead cells were stained by SYTOX Orange. **K** CAV1 overexpression cells were treated with 0 or 2 mM Na-OHB, and lipid ROS levels within cells were measured by flow cytometry. **L** CAV1 overexpression cells were treated with 0 or 2 mM Na-OHB, and intracellular Fe^2+^ levels were assessed using flow cytometry. **A** Data were shown as the mean ± SD, *n* = 3, two-tailed *t-*test or one-way ANOVA with Dunnett *t*-test. **B**, **C**, **E**–**L** Data were shown as the mean ± SEM, *n* = 3, two-tailed *t*-test or one-way ANOVA with Dunnett *t*-test. *P < 0.05, **P < 0.01, ***P < 0.001, and ****P < 0.0001.

When PANC1 and MIA PaCa-2 cells were treated with gradient concentrations of Na-OHB, the expression of CAV1 was downregulated at the protein level (Fig. 5A), but its transcriptional changes were not entirely consistent (Fig. 5B). Simultaneously, the gradient concentrations of Na-OHB also induced tumor cell death, and the proportion of dead cells increased with higher Na-OHB concentrations (Fig. 5C and Supplementary Fig. 7A). Upon further investigation utilizing 2 mM Na-OHB treatment, an increase in the proportion of dead cells compared to the control group was observed without a corresponding increase in the proportion of apoptotic cells (Fig. 5D). To further confirm that the form of cell death induced by Na-OHB is ferroptosis, we introduced the ferroptosis inhibitor Ferrostatin-1 (Fer-1) in the experimental group. The results indicated that Fer-1 inhibits cell death caused by Na-OHB (Fig. 5E and Supplementary Fig. 7B). At the same time, we found that Na-OHB induced an overload of Fe^2+^ and an accumulation of excessive lipid ROS within the cells, while the cellular antioxidant system, including GSH and its precursor cystine, showed a certain degree of decline. These characteristic cellular changes associated with ferroptosis were reversed under the treatment of Fer-1 (Fig. 5F–I). These findings suggest that the cell death induced by Na-OHB in pancreatic cancer cells may be attributed to ferroptosis. However, cell lines overexpressing CAV1 demonstrated resistance to Na-OHB-induced ferroptosis when treated with 2 mM concentrations. No increase in cell death was observed (Fig. 5J), and there were no significant changes in intracellular Fe^2+^ levels nor lipid ROS levels (Fig. 5K, L).

### CAV1 transcriptionally activates ferroptosis-related genes through the AMPK/NRF2 pathway and stabilizes SLC7A11

**Fig. 6:**
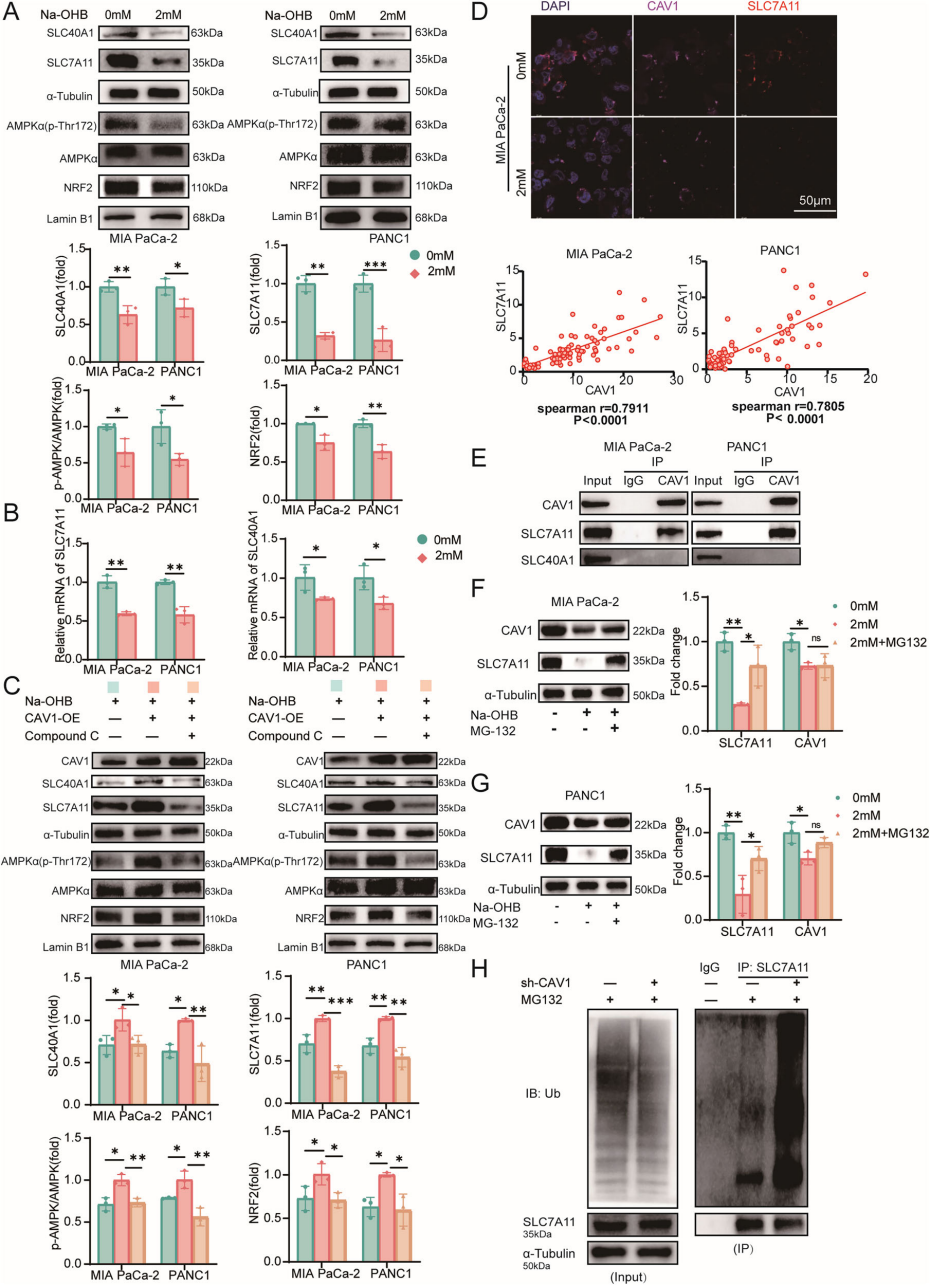
CAV1 transcriptionally activates ferroptosis-related genes through the AMPK/NRF2 pathway and stabilizes SLC7A11. **A** Western blotting of proteins in the AMPK/NRF2 pathway after treatment by Na-OHB. **B** qPCR of *SLC40A1* and *SLC7A11* after treatment by Na-OHB. **C** Western blotting of proteins in AMPK/NRF2 pathway. **D** Representative immunofluorescence staining of MIA PaCa-2 after being treated by Na-OHB with CAV1 in magenta, SLC7A11 in red, and DAPI in blue (upper), and correlation scatter plot of CAV1 and SLC7A11(lower). **E** Co-IP of CAV1 and SLC7A11, SLC40A1 in MIA PaCa-2(left) and PANC1(right) cells. **F** Western blotting of CAV1, SLC7A11, and α-Tubulin after being treated by Na-OHB with or without MG-132 in MIA PaCa-2. **G** Western blotting of CAV1, SLC7A11, and α-Tubulin after being treated by Na-OHB with or without MG-132 in PANC1. **H** IP of SLC7A11 in MIA PaCa-2 cells and ubiquitin was detected. **A**, **C**, **F**, **G** Data were shown as the mean ± SD, *n* = 3, two-tailed *t*-test or one-way ANOVA with Dunnett *t*-test. **B** Data were shown as the mean ± SEM, *n* = 3, two-tailed *t*-test. **D** Spearman correlation analysis. *P < 0.05, **P < 0.01, ***P < 0.001, and ****P < 0.0001.

As reported previously, CAV1 activates the AMPK/NRF2 signaling pathway, promoting the expression of ferroptosis-related genes. NRF2 downstream regulatory genes include the Fe^2+^ pump SLC40A1 and the cystine transporters SLC7A11. To investigate whether Na-OHB-induced intracellular peroxidation and dysregulation of Fe^2+^ levels are also regulated through this signaling pathway, we examined the activation levels of the AMPKα, NRF2 protein content in the nucleus, and key downstream molecules after Na-OHB treatment. The results demonstrated a reduction in p-AMPK/AMPK levels and NRF2 protein content in the nucleus, leading to a significant decrease in the transcriptional levels and protein contents of SLC40A1 and SLC7A11 (Fig. 6A, B). Further validation using CAV1-overexpressing cell lines and the AMPKα inhibitor Compound C confirmed that CAV1 overexpression counteracts Na-OHB-induced suppression of AMPK/NRF2 signaling, while Compound C alleviates this effect without affecting the protein level of CAV1 (Fig. 6C). Besides, immunofluorescence staining showed that CAV1 expression was significantly correlated with the expression of SLC7A11, with correlation coefficients of 0.7805 and 0.7911 in the two cell lines, respectively (Fig. 6D). The co-IP experiment demonstrated an interaction between CAV1 and SLC7A11, whereas no such interaction was observed with SLC40A1 (Fig. 6E). To further investigate whether the interaction between CAV1 and SLC7A11 affects the stability of SLC7A11, we employed the proteasome inhibitor MG-132. The addition of MG-132 was found to partially reverse the decrease in SLC7A11 protein levels (Fig. 6F, G), indicating that CAV1 not only activates the transcription of SLC7A11 but may also contribute to its protein stability. Consequently, we assessed the ubiquitination levels of SLC7A11 in CAV1-knockdown cells. The results revealed a significant increase in SLC7A11 ubiquitination upon CAV1 knockdown (Fig. 6H and Supplementary Fig. 7C). These findings demonstrate that CAV1 not only regulates the transcription of both SLC40A1 and SLC7A11 but also plays a crucial role in stabilizing the SLC7A11 protein, and consistent results were obtained in Pan02 cells (Supplementary Fig. 7D–J).

### CAV1 levels modulate ferroptosis sensitivity in pancreatic cancer cells

To investigate the direct impact of CAV1 on ferroptosis, we assessed the levels of SLC7A11 in CAV1-overexpressing and CAV1-knockdown cell lines. Consistent with the aforementioned results, the expression of SLC7A11 at both the transcriptional and protein levels was found to correlate with changes in CAV1 (Supplementary Fig. 8A–E). Using the classic ferroptosis inducer RSL3, we compared the sensitivity of wild-type, CAV1-overexpressing, and CAV1-knockdown pancreatic cancer cell lines to RSL3. The results showed that although the wild-type, CAV1-knockdown, and CAV1-overexpressing conditions exhibited minimal levels of cell death in the absence of RSL3 supplementation, overexpression of CAV1 enhanced the cells’ protective mechanisms against ferroptosis, leading to increased resistance to RSL3. On the other hand, reducing CAV1 expression diminishes these protective mechanisms, making the cells more susceptible to RSL3-induced ferroptosis (Supplementary Fig. 8F). Considering that the global knockdown or overexpression of CAV1 in this context did not directly induce ferroptosis, we investigated whether the cells exhibited potential, yet insufficient, functional alterations that could enable them to resist or rapidly respond to inducer-induced ferroptosis. The results showed that CAV1-knockdown cells had elevated levels of lipid ROS (Supplementary Fig. 8G) and decreased mitochondrial membrane potential and mitochondrial mass, whereas CAV1-overexpressing cells did not differ significantly from the wild type in these respects (Supplementary Fig. 8H). These results suggest that knocking down CAV1 may reduce the reserve of cellular redox homeostatic capacity, which in turn elevates the sensitivity of pancreatic cancer cells to the induction of ferroptosis.

### Ketogenic diet reduces CAV1 and SLC7A11 expression and tumor burden in tumor-bearing mouse models

**Fig. 7:**
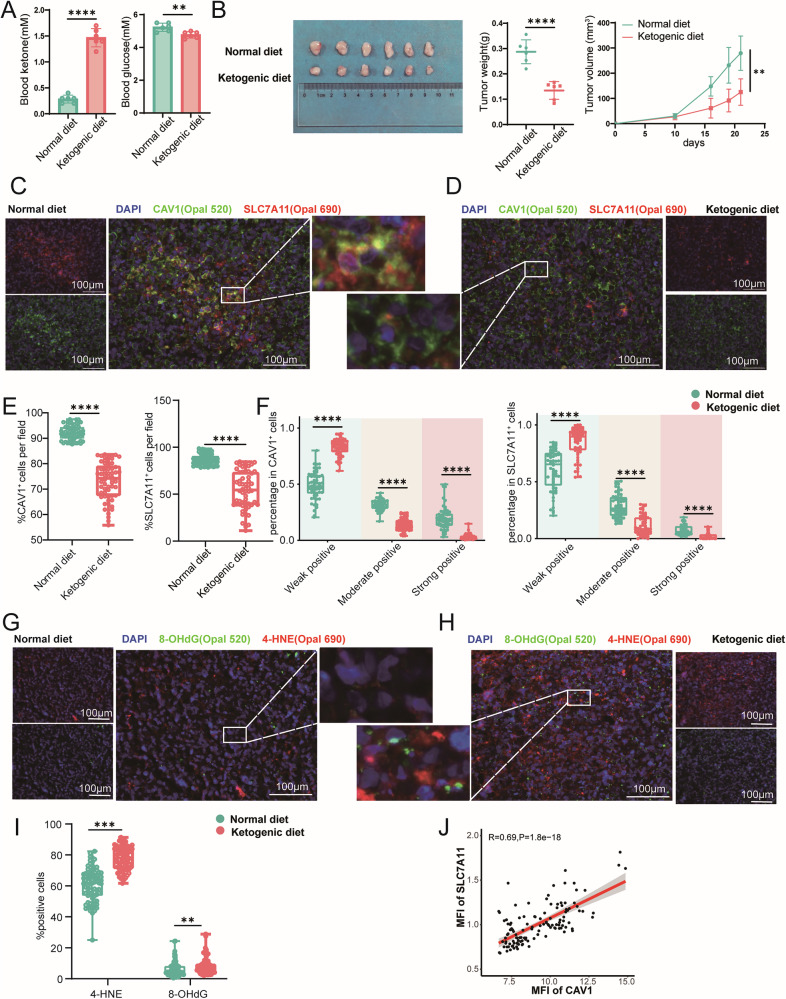
Ketogenic diet reduces CAV1 and SLC7A11 expression and tumor burden in MIA PaCa-2 model. **A** Blood ketone (left) and glucose (right) levels of normal or ketogenic diet nude mice bearing MIA PaCa-2 xenograft tumor. **B** Tumor weight and volume of normal or ketogenic diet nude mice bearing MIA PaCa-2 xenograft tumor. **C** Representative image of multiplex immunofluorescence staining for CAV1 (green) and SLC7A11 (red) on MIA PaCa-2 model fed with a normal diet. **D** Representative image of multiplex immunofluorescence staining for CAV1 (green) and SLC7A11 (red) on MIA PaCa-2 model fed with a ketogenic diet. **E** Percentage of CAV1^+^ (left) or SLC7A11^+^ (right) cells per field on MIA PaCa-2 xenograft tumor tissue sections. **F** Percentage of gradient CAV1^+^ (left) or SLC7A11^+^ (right) cells on MIA PaCa-2 xenograft tumor tissue sections. **G** Representative image of multiplex immunofluorescence staining for 8-OHdG (green) and 4-HNE (red) on MIA PaCa-2 model fed with a normal diet. **H** Representative image of multiplex immunofluorescence staining for 8-OHdG (green) and 4-HNE (red) on MIA PaCa-2 model fed with a ketogenic diet. **I** Percentage of 4-HNE^+^ (left) or 8-OHdG^+^ (right) cells per field on MIA PaCa-2 xenograft tumor tissue sections. **J** Correlation scatter plot of mean fluorescence intensity (MFI) between CAV1 and SLC7A11 on MIA PaCa-2 xenograft tumor tissue sections. **A**, **B**, **E**, **F**, **I** Data were shown as the mean ± SD, *n* = 6, two-tailed *t*-test or two-way ANOVA. **J** Spearman correlation analysis. **P < 0.01, ***P < 0.001, and ****P < 0.0001.

**Fig. 8:**
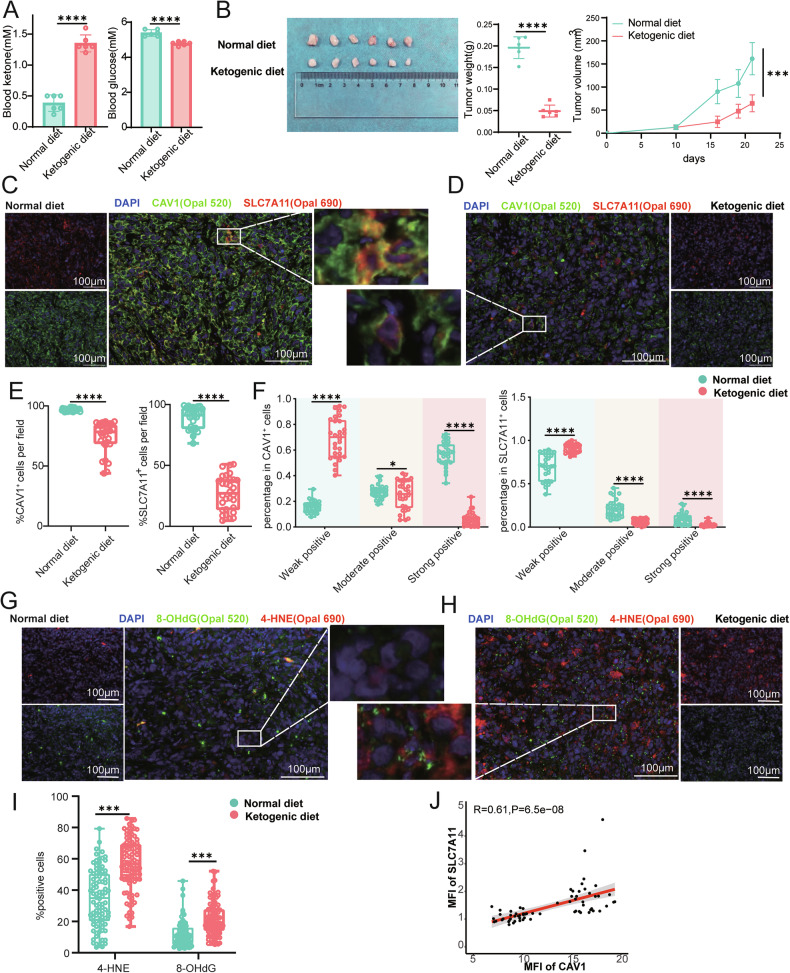
Ketogenic diet reduces CAV1 and SLC7A11 expression and tumor burden in PANC1 model. **A** Blood ketone (left) and glucose (right) levels of normal or ketogenic diet nude mice bearing PANC1 xenograft tumor. **B** Tumor weight and volume of normal or ketogenic diet nude mice bearing PANC1 xenograft tumor. **C** Representative image of multiplex immunofluorescence staining for CAV1 (green) and SLC7A11 (red) on PANC1 model fed with a normal diet. **D** Representative image of multiplex immunofluorescence staining for CAV1 (green) and SLC7A11 (red) on PANC1 model fed with a ketogenic diet. **E** Percentage of CAV1^+^ (left) or SLC7A11^+^ (right) cells per field on PANC1 xenograft tumor tissue sections. **F** Percentage of gradient CAV1^+^ (left) or SLC7A11^+^ (right) cells on PANC1 xenograft tumor tissue sections. **G** Representative image of multiplex immunofluorescence staining for 8-OHdG (green) and 4-HNE (red) on PANC1 model fed with a normal diet. **H** Representative image of multiplex immunofluorescence staining for 8-OHdG (green) and 4-HNE (red) on PANC1 model fed with a ketogenic diet. **I** Percentage of 4-HNE^+^ (left) or 8-OHdG^+^ (right) cells per field on PANC1 xenograft tumor tissue sections. **J** Correlation scatter plot of MFI between CAV1 and SLC7A11 on PANC1 xenograft tumor tissue sections. **A**, **B**, **E**, **F**, **I** Data were shown as the mean ± SD, *n* = 6, two-tailed *t*-test or two-way ANOVA. **J** Spearman correlation analysis. **P < 0.01, ***P < 0.001, and ****P < 0.0001.

To assess the impact of ketone bodies on the CAV1 molecule in vivo, we established three tumor-bearing mouse models subjected to either a ketogenic diet or a normal diet. The ketogenic diet markedly elevated blood ketone concentrations in the tumor-bearing mice, increasing from baseline levels of less than 0.5 mM to ~1.2 mM, while inducing a marginal reduction in blood glucose levels (Fig. 7A). After three weeks, the ketogenic diet significantly diminished tumor burden and impeded tumor progression in the mice (Fig. 7B). Further analysis involved performing multiplex immunofluorescence staining on mouse tumor tissue sections. The results showed that tumors from mice fed with a normal diet exhibited high expression of CAV1 and SLC7A11, with a positive rate of around 90% for both molecules (Fig. 7C). Additionally, there was significant colocalization of these two molecules on the cell membrane. In contrast, tumors from mice fed with a ketogenic diet showed a marked decrease in CAV1 and SLC7A11 protein, with a reduced positive rate compared to the normal diet group (Fig. 7D, E). Statistical analysis of the fluorescence intensity at the single-cell level revealed that, following the ketogenic diet, the proportion of cells with low expression of CAV1 and SLC7A11 increased among CAV1^+^ or SLC7A11^+^ cells. Conversely, the proportion of cells with medium to high expression of CAV1 and SLC7A11 significantly decreased (Fig. 7F). These consistent results were observed in both the PANC1 xenograft model in nude mice and the Pan02 tumor model in C57 mice (Fig. 8A–F and Supplementary Fig. 9A–F). Meanwhile, tumor tissues of ketogenic diet mice showed elevated levels of markers of ferroptosis, such as 4-HNE and 8-OHdG (Fig. 7G–I), which was consistent in the other two models (Fig. 8G–I and Supplementary Fig. 9H–J). Correlation analysis of CAV1 molecules and SLC7A11 showed a significant positive correlation between them, with correlation coefficients of 0.69, 0.61, and 0.88 in the three tumor models, respectively (Figs. 7J, 8J and Supplementary Fig. 9G). In summary, the ketogenic diet led to a significant decrease in both the expression of CAV1 and SLC7A11 at the single-cell level and their overall positive rate in tumor tissues, which also significantly reduced the tumor burden through inducing ferroptosis in tumor-bearing mice.

## Discussion

In this study, we investigated the molecular mechanisms that connect ketogenic metabolism and ferroptosis in pancreatic cancer. Our research identified CAV1 as a crucial molecule that acts as a central link between these two processes. We utilized a comprehensive array of analytical techniques, including differentially expressed gene analysis, WGCNA, univariate Cox regression, Kaplan–Meier survival analysis, and LASSO regression. Through these methods, we identified an eight-gene list associated with ketogenic metabolism and a seven-gene list related to ferroptosis. The ROC curves demonstrated that both gene lists exhibited favorable prognostic indications for pancreatic cancer. Subsequently, two phenotypic scores were calculated, and both scores were elevated in PAAD compared to normal tissues. The evaluation of immune infiltration showed a high degree of concordance in the types of infiltrating immune cells. The PPI network highlighted CAV1 as a central component within this network. scRNA-seq data analysis revealed a heterogeneous cellular distribution of CAV1 and the two phenotypes. CAV1 expression was predominantly localized within tumor cells, with both phenotypic scores of these cells surpassing those observed in other cell types. In vitro experiments utilizing Na-OHB as a ketone body source confirmed the mechanism through which ketone bodies induce ferroptosis via the downregulation of the CAV1/AMPK/NRF2 pathway. Furthermore, by manipulating the CAV1 molecule at the cellular level, we assessed the role of the CAV1 in regulating ferroptosis and its critical protective molecule, SLC7A11. The knockdown of CAV1 resulted in the accumulation of intracellular lipid peroxides, which reduced the cellular reserve capacity against oxidative stress. In addition, CAV1 interacts with SLC7A11 and affects the cellular protective potential of SLC7A11 from both transcriptional and proteasomal degradation pathways. Utilizing mouse tumor models, we conducted an in-depth evaluation of the ferroptosis status and the expression changes of CAV1 and SLC7A11 at the single-cell level within tumor tissues. This analysis substantiates the conclusion that CAV1 functions as a pivotal bridging molecule connecting ketone metabolism and ferroptosis.

Elevated levels of β-hydroxybutyrate serve as a serological hallmark of the ketogenic diet, which plays a crucial role in the therapeutic management of various diseases [19, 21]. Multiple studies have reported varying effects of the ketogenic diet on cancer, with some suggesting promotion and others indicating inhibition [22–25]. These discrepancies may arise from variations in tumor types, serum ketone body levels, and the cachectic state of patients with advanced-stage cancer. Therefore, the application of the ketogenic diet or β-hydroxybutyrate in tumor treatment necessitates a case-specific evaluation, as no universal consensus has been established. Potential mechanisms in tumor treatment include the reduction of glucose availability [26], inhibition of cellular proliferation [27], and suppression of inflammation-induced cancer transformation [28, 29]. The lysine β-hydroxybutyrylation may represent a viable therapeutic target [30, 31]. Additionally, in mouse models of pancreatic cancer, a systemic ketogenic state induces lipid peroxidation and disrupts redox homeostasis, thereby facilitating tumor ferroptosis [19]. This study establishes a link between the anticancer effects of a ketogenic diet and ferroptosis, highlighting the need for further investigation into additional underlying mechanisms. Our research has enhanced the comprehension of the interplay between ketogenic and ferroptosis phenotypes through pathways associated with fatty acid metabolism and caveola cellular components. Furthermore, this study offers a theoretical basis for the induction of ferroptosis in pancreatic cancer cells through a ketogenic diet, thereby contributing to the control of pancreatic cancer progression.

CAV1 serves as a vital structural protein within caveolae [32]. Considering the remarkable variability in CAV1 expression across different cell types, alterations in these cell types may reflect adaptive changes in tumorigenesis or therapeutic effects under stressful conditions. High CAV1 expression in tumor cells and low expression in the stroma are associated with poor prognosis and tumor progression [33, 34]. In cancer-associated fibroblasts, CAV1 was significantly downregulated [35, 36] accompanied by a significant upregulation of ketogenesis-related genes, reflecting an intrinsic connection between CAV1 and ketone metabolism in fibroblasts [37]. Our analysis indicates that fibroblasts demonstrate an enhanced ketogenic phenotype. The ketone bodies produced through fibroblast metabolism can serve as nutrient substrates for tumor cells [38, 39]. This hypothesis is reasonable within the framework of tumorigenesis. Nonetheless, the ketogenesis prompted by this fibroblast-tumor cell interaction might result in consistently low concentrations of ketone bodies, which could partially elucidate the limited efficacy of certain ketogenic therapies. It is posited that moderate-to-high levels of ketone bodies may be necessary to induce substantial intracellular environmental disruption, ultimately leading to tumor cell death. The correlation between CAV1 and ketogenic metabolism in tumor cells requires further study. Our study proposes a new perspective, suggesting that elevated ketone body levels downregulate CAV1. According to previous studies, CAV1 attenuates the accumulation of excess intracellular Fe^2+^ through activation of the ferritin light chain/ferritin heavy chain pathway, which in turn protects from Fenton reaction-induced oxidative stress damage [40]. In addition, CAV1 activated the AMPK/NRF2 signaling pathway, increased Fpn expression [41, 42], and pumped out excess intracellular Fe^2+^. In our study, Na-OHB induces ferroptosis in tumor cells by downregulating the genes regulated by CAV1/AMPK/NRF2 and affecting the interaction between CAV1 and SLC7A11. On the other hand, as demonstrated in functional enrichment and correlation analyses, CAV1 may alter the immune cell types in the tumor microenvironment through modulation of the TGF-β signaling pathway. Additionally, CAV1 could impact the metabolic demands of tumor cells by influencing the uptake and metabolism of fatty acids.

The cystine/glutamate antiporter SLC7A11 serves as an inhibitor of ferroptosis [43]. Research indicates that the downregulation of CAV1 is accompanied by ferroptosis in autoimmune hepatitis [44]. Moreover, the absence of CAV1 exacerbated the susceptibility to ConA-induced hepatitis by decreasing SLC7A11 levels. CAV1 can mitigate autophagy-dependent ferroptosis, which may consequently reduce calcium oxalate stone formation in urolithiasis [45]. In addition, CAV1 has been shown to alleviate diabetes-associated cognitive impairment by modulating ferroptosis [42]. In fibrotic diseases, elevated expression of CAV1 is instrumental in inducing ferroptosis, thereby exerting its anti-fibrotic effects [46, 47]. High levels of CAV1 inhibit ferroptosis in head and neck squamous cell carcinoma [48]. Our study revealed that akin to the protective mechanisms against liver damage, the downregulation of CAV1 also caused the downregulation of SLC7A11 transcription and increased the degradation of the proteasome pathway, thereby facilitating the process of ferroptosis in these cells.

Our analysis elucidates the complex interplay between ketogenic metabolism and ferroptosis, highlighting the mediating function of CAV1. Further exploration of these molecular mechanisms could yield significant insights into the unique characteristics of pancreatic cancer at the single-cell level and inform potential therapeutic strategies. Importantly, this study may enhance the theoretical framework supporting the adjuvant use of a ketogenic diet in the treatment of pancreatic cancer, thereby offering renewed hope for patients with pancreatic cancer.

Our study also has the following limitations. The data from the PAAD dataset in TCGA and the normal pancreatic tissue data from the GTEx database were not perfectly matched, unlike other cancer types. This discrepancy may have introduced a slight bias due to differences in the sample sources.

## Materials and methods

Supplementary Fig. 1 presents the detailed process of this study.

### Data collection and preprocessing

RNA-sequencing data consisting of 178 tumor samples and four normal samples with clinical information, including survival duration and pathological grades for PAAD, were obtained from the TCGA database (https://portal.gdc.cancer.gov/). RNA-sequencing data for 167 normal pancreatic tissues from the Genotype-Tissue Expression Project (GTEx) database were downloaded from the University of California Santa Cruz Xena (http://xena.ucsc.edu/). Additionally, scRNA-seq data (GSE41017) were acquired from the Gene Expression Omnibus (GEO) database (https://www.ncbi.nlm.nih.gov/geo/). Ketogenic metabolism-related pathways were retrieved from the GSEA database (https://www.gsea-msigdb.org/gsea/index.jsp) (Supplementary Table 1), and the genes within these pathways were combined to obtain a list of 423 ketone-related genes (Supplementary Table 2). Ferroptosis-related genes were obtained from the FerrDb database [49], and 218 nonredundant human genes were selected [50] (Supplementary Table 3).

### DEGs identification

R (4.1.2) software was used to integrate the mRNA expression matrix of PAAD and normal pancreatic tissues. DEGs were identified by the “DESeq2” R package [51] with an adjusted *P* < 0.05 and |log2FC| ≥1.

### Gene co-expression network construction

By clustering genes that exhibit similar expression profiles into modules, WGCNA facilitates the analysis of how gene co-expression networks correlate with particular features [52]. This process was performed using the “WGCNA” R package, incorporating DEGs in the analysis. The “Hclust” function was applied to achieve hierarchical clustering and to pinpoint any distinct outliers. The gene expression relationship was adjusted to align with a scale-free network architecture by employing the “pick Soft Threshold” function. Then, the gene co-expression matrix was transformed into an adjacency matrix through the “adjacency” function, utilizing the chosen thresholding power β. This adjacency matrix was then converted into a topological overlap matrix. Finally, the identification of modules was achieved using hierarchical clustering alongside the dynamic shear tree method, with Pearson’s correlation analysis being employed to uncover modules that correlate with the TNM stage.

### Screening of ketone-stage-survival DEGs and ferroptosis-stage-survival DEGs

Venn diagrams [53] were used to define ketone-stage DEGs and ferroptosis-stage DEGs. Kyoto Encyclopedia of Genes and Genomes (KEGG) and Gene Ontology (GO) enrichment analyses were conducted to pinpoint closely associated biological functions. Then, selected genes were assessed by univariate Cox regression analysis and Kaplan–Meier curves, employing the “survival” R package, to investigate their correlation with patients’ overall survival rates. Genes *P* < 0.05 and the hazard ratio (HR) are shown in the forest map. In light of the rigorous scrutiny applied to genetic selection in both survival-related analyses, we set the inclusion criterion to *P* < 0.1 for ketone-stage-survival DEGs and ferroptosis-stage-survival DEGs.

### Phenotype scoring for ketogenic metabolism and ferroptosis

Least absolute shrinkage and selection operator (LASSO) regression was conducted through the “glmnet” R package [54]. Genes that passed through this filtering process were used to develop prognostic models and to calculate risk scores for individual samples. The efficacy of these models was validated using ROC curves and Kaplan–Meier analysis. The ssGSEA function built-in “GSVA” R package [55] was utilized to compute the ketogenic phenotype and ferroptosis phenotype scores for each sample. The Mann-Whitney U test was utilized to assess the differences in phenotypic scores amongst tumor and normal pancreatic tissues, while Pearson’s correlation analysis was applied to evaluate the association between them.

### Immune infiltration analysis

The CIBERSORT algorithm [56] was applied to assess the immune infiltration status utilizing 22 immune cells’ characteristic gene set. The Kruskal-Wallis test was used to compare tumor and pancreatic tissues. Then, Spearman correlation analysis was used to explore the correlation between the infiltration of immune cells with non-zero abundance in more than half of the cells.

### PPI establishment and identification of key genes

The PPI network of genes screened by univariate Cox analysis (*P* < 0.1) was constructed using the STRING online tool (https://string-db.org/) with an interaction score threshold of 0.7. The constructed network was visualized and analyzed using Cytoscape software v3.7.1. The CytoHubba plugin [57] in Cytoscape software was used to evaluate the degree and Maximum Neighborhood Component. The MCODE plugin was used to detect dense regions in the network as subnetworks. The ClueGO plugin [58] was used for the GO enrichment of selected genes.

### scRNA-seq data processing

The scRNA-seq data numbered GSE141017 was processed according to the “Seurat” R package [59]. Briefly, high-quality cells were filtered based on mitochondrial gene content, ribosomal gene content, number of gene expressions per cell, and number of counts. Then, the cells were clustered unsupervised by linear/nonlinear dimensionality reduction methods such as PCA, UMAP, and tSNE, and the cell type of each cluster was defined concerning the “SingleR” R package [60]. Next, the ketogenic and ferroptosis scores for individual cells were calculated using the AddModuleScore function and previously acquired phenotypic genes.

### Cell culture and treatment

PANC1 and Mia PaCa-2 pancreatic cancer cell lines were acquired from the American Type Culture Collection (ATCC, United States). The mouse pancreatic cancer cell line Pan02 was a gift from the Department of Hepatobiliary Surgery, Xi-Jing Hospital, Fourth Military Medical University. Cells were cultured at 37 °C with 5% CO_2_ in Dulbecoo’s modified Eagle’s medium or RPMI-1640 medium supplemented with 10% fetal bovine serum. Sodium 3-hydroxybutyrate (Na-OHB, Aladdin, 150-83-4) was diluted to 0, 1, 2, and 5 mM. The pancreatic cancer cell lines were seeded into six-well plates at a density of 2 × 10^5^ cells/well and cultured overnight, after which Na-OHB, Ferrostatin-1 (Fer-1, MCE, HY-100579), MG-132 (MCE, HY-13259), or Compound C (MCE, HY-13418A) at the indicated concentrations was added to the wells. After 72 h of induction, the cells were collected for subsequent experiments.

### Evaluation of cell death, apoptosis, lipid ROS, and Fe^2+^

After induction, the cells were stained with Hoechst 33342 (Beyotime, C1022) for all cells and SYTOX Orange (Invitrogen, S34861) for dead cells, photos were then captured by fluorescence microscopy, and ImageJ software was used to count cells automatically. Cell apoptosis was detected with Annexin V-FITC (Keygen, KGA107) and 7-AAD viability staining solution (Biolegend, 420403). A lipid peroxidation sensor (BODIPY 581/591 C11, Invitrogen, D3861) was used to detect lipid ROS, and FerroOrange (MKBio, MX4559) was used to detect Fe^2+^ in the cells. These staining procedures were conducted according to the product manuals, and lipid ROS levels were calculated by the mean fluorescence intensity of FITC/PE.

### Evaluation of GSH/GSSG and cystine

GSH and GSSG Levels were measured using a GSH and GSSG Assay Kit (Beyotime, S0053) according to the product manual. Briefly, the absorbance values at A412 were measured for the same sample under two conditions: before and after the removal of GSH, following its reaction with the detection reagent. The concentrations of total glutathione and GSSG were calculated based on the standard curve, and the concentration of GSH was calculated as the formula: GSH=Total Glutathione-GSSG×2, GSH/GSSG was used for statistical analysis. The cystine levels were measured using the BioTracker Cystine-FITC Live Cell Dye (Sigma-Aldrich, SCT047). After treatment, the processed live cells were collected and stained, and the mean fluorescence intensity was quantified using FACS.

### Western blot, immunoprecipitation (IP), and co-immunoprecipitation (Co-IP)

Cells were harvested using an ice-cold radioimmunoprecipitation assay buffer containing a 1% Halt protease and phosphatase inhibitor cocktail. Cell lysates were centrifuged at 14,000 rpm for 15 min at 4 °C, after which the supernatants were collected as total protein. The nuclear protein was extracted by Nuclear and Cytoplasmic Protein Extraction Kit (Beyotime, P0028). The protein concentrations were quantified using the BCA method. After protein separation via 10% SDS-PAGE, the proteins were transferred onto 0.45 μM PVDF membranes (Millipore, IPVH00010) and blocked with 5% skim milk at room temperature for 1 h. Membranes were incubated with the following primary antibodies: anti-Caveolin-1 Monoclonal Antibody (Proteintech, 66067-1, 1:2000), anti-SLC7A11 (Abcam, ab118470, 1:1000), anti-SLC40A1 (Proteintech, 26601-1-AP, 1:1000), anti-AMPKα (p-Thr172) (abMart, TP56027, 1:1000), anti-AMPKα (Proteintech 10929-2-AP, 1:1000), anti-NRF2 (Proteintech, 80593-1-RR, 1:1000), anti-Lamin B1 (Proteintech, 66095, 1:10,000), anti-ubiquitin (Proteintech, 80992-1-RR, 1:10,000), and anti-α-tubulin (Proteintech, 66031-1, 1:10,000) overnight at 4 °C. Following this, membranes were washed and incubated with goat anti-mouse IgG (H + L) secondary antibody, HRP (Pierce, 31430, 1:10,000), or goat anti-rabbit IgG (H + L) secondary antibody, HRP (Invitrogen, 31460, 1:10,000). The immune complexes were visualized by an enhanced chemiluminescence kit (Engreen, 29050).

IP and Co-IP were conducted utilizing the Pierce Co-Immunoprecipitation Kit (Thermo Fisher Scientific, 26149) following the manufacturer’s instructions. Briefly, cells were lysed using a mild lysis buffer, and the protein lysates were added to a 5 µg primary antibody-coated column and incubated overnight at 4 °C with gentle rotation. The samples were then washed with coupling buffer and eluted using elution buffer. The eluted products were mixed with loading buffer and subjected to WB analysis.

Full-length uncropped original western blots are shown in Supplementary Material 2.

### Quantitative real-time PCR (qRT-PCR)

RNA was extracted by Total RNA Kit II (OMAGA-BIO-TEK, D6934-01). Reverse transcription was performed by PrimeScript™ RT Master Mix (Perfect Real Time) (TaKaRa, RR036A). qRT-PCR was performed with TB Green® Premix Ex Taq™ II (Tli RNase H Plus) (TaKaRa, RR820A). The qRT-PCR primer sequences were listed as follows: hβ-ACTIN: forward primer-5′-CATGTACGTTGCTATCCAGGC-3′, reverse primer-5′-CTCCTTAATGTCACGCACGAT-3′; hCAV1: forward primer-5′-GCGACCCTAAACACCTCAAC-3′, reverse primer-5′-ATGCCGTCAAAACTGTGTGTC-3′; hSLC7A11: forward primer-5′-TCTCCAAAGGAGGTTACCTGC-3′, reverse primer-5′-AGACTCCCCTCAGTAAAGTGAC-3′; hSLC40A1: forward primer-5′- CTACTTGGGGAGATCGGATGT-3′, reverse primer-5′-CTGGGCCACTTTAAGTCTAGC-3′.

### Immunofluorescence

For immunofluorescence staining, following a 15-min fixation in 4% paraformaldehyde, cells underwent PBS washes. Blocking was performed using 5% goat serum in PBS, after which cells were subjected to incubation at 4 °C overnight with primary antibodies: anti-Caveolin-1 (Proteintech, 66067-1, 1:500) and anti-SLC7A11 (Abcam, ab118470, 1:500) antibodies. After that, the cells were thrice washed in PBS and stained with Alexa Fluor 555 donkey anti-rabbit IgG (H + L) (Invitrogen, 1:200) and goat anti-mouse IgG (H + L) DyLight 594 (Invitrogen, 1:200) secondary antibodies at room temperature for 1 h. DAPI (Beyotime) was utilized for nuclear staining. Confocal microscopy (LEICA) was employed to capture images of the stained cells.

### Construction of stable knockdown and overexpression cell lines

MIA PaCa-2 and PANC1 cells were transfected using shCAV1 or CAV1-overexpressing lentiviruses. The multiplicity of infection (MOI) was set at 10 for MIA PaCa-2 cells and 100 for PANC1 cells. Cultural mediums were changed 24 h post-transfection, followed by monitoring of the green fluorescent protein signal. Stable transfectants were selected 48 h later using puromycin at a concentration of 4 µg/mL. The shRNA sequence is: 5′-CCAGAAGGGACACACAGTTTC-3′, and the transcript for overexpression has the GenBank accession number NM_001753.5. Stable overexpression, knockdown, and wild-type cells were treated with 0, 0.5, 1, 2, and 4 μM RSL3 for 24 h. Subsequently, the cells were stained and imaged.

### Mitochondrial membrane potential and mass detection

The mitochondrial membrane potential of cells was assessed using Mito-Tracker Deep Red FM (Beyotime Biotech, China, C1032), while mitochondrial mass was evaluated with Mito-Tracker Green (Beyotime Biotech, China, C1048). Both parameters were quantified using flow cytometry.

### Mouse model

Male or female nude mice at 8 weeks old were subcutaneously inoculated with either 5 × 10^6^ MIA PaCa-2 cells or 5 × 10^6^ PANC1 cells. Visible tumors were observed after 10 days. C57BL/6 mice at 8 weeks old were subcutaneously inoculated with 1 × 10^6^ Pan02 cells. Visible tumors were observed after 7 days. Based on tumor size, the mice were evenly divided into two groups. One group was maintained on a normal diet (XIETONG SHENGWU, 1010084), while the other group was transitioned to a ketogenic diet (XIETONG SHENGWU, XTKD01) over one week. Tumor dimensions were measured every 4 days using calipers. Tumor volume was calculated using the following formula: Tumor volume = (length × width^2^)/2. Three weeks after grouping, blood ketone levels were measured, and the mice’s weights were recorded. Subsequently, the tumor-bearing nude mice were euthanized, and the subcutaneous tumors were excised, weighed, and photographed. The excised tumors were immediately fixed in 4% paraformaldehyde, processed for paraffin embedding, sectioned, and subjected to multiplex immunofluorescence staining.

### Multiplex immunofluorescence staining

Deparaffinization of tissue sections to water, followed by antigen retrieval using citrate buffer (pH = 6) or Tris-EDTA (pH = 9). After blocking with 5% goat serum, sections were incubated with primary antibodies against anti-Caveolin-1 (Proteintech, 66067-1, 1:1000), anti-SLC7A11 (Abcam, ab118470, 1:1000), anti-CK18 (Abcam, ab181597, 1:2000), anti-8-OHdG (Abcam, ab48508, 1:200), and anti-4-HNE (Abcam, ab46545, 1:500). Post-primary antibody incubation, a universal secondary antibody and TSA fluorescence amplification (AKOYA BIOSCIENCES, SKU NEL810001KT) were used to enhance the fluorescent signals. Subsequently, nuclei were stained with DAPI, and sections were mounted with neutral resin. Images were captured using PerkinElmer’s Vectra® Polaris™ Quantitative Pathology Workstation, and analysis was conducted using inForm software.

### Statistical analyses

Statistical analyses of the bioinformatics data were performed using R (4.1.2) software. Data were visualized using the R package “ggplot2” [61]. *P* < 0.05 was considered statistically significant unless otherwise stated. Statistical analyses in the experimental part were performed using the GraphPad Prism 8.0 software. *P* values were determined using a Student’s two-tailed *t*-test, spearman correlation analysis, logistic regression analysis, or one-way ANOVA, and data were presented as mean ± SD or mean ± SEM. The significance level is denoted as ns (not significant), *P < 0.05, **P < 0.01, ***P < 0.001, and ****P < 0.0001.

## Supplementary information

Supplementary Figures and Tables

Supplementary figure 1: Flow chart of this study.

Supplementary figure 2: Sample clustering diagram of WGCNA.

Supplementary figure 3

Supplementary figure 4

Supplementary figure 5

Supplementary figure 6

Supplementary figure 7

Supplementary figure 8

Supplementary figure 9

Western blotting

## Data Availability

The code and original data are available upon request.
