# Supplementary material for: Integrative insights into the role of CAV1 in ketogenic diet and ferroptosis in pancreatic cancer
Source: Cell Death Discov. 2025 Apr 4;11:139. doi: 10.1038/s41420-025-02421-z (PMC11968908; doi:10.1038/s41420-025-02421-z)
Supplement: Supplementary file 1 — Supplementary Figures and Tables [file 41420_2025_2421_MOESM1_ESM.docx]

***Supplementary Material 1***

**Supplementary Figures and Tables**

**Supplementary Tables**

**Supplementary table 1:**

Pathways associated with ketone body metabolism in GSEA

| **Source** | **Pathway name** |
| --- | --- |
| GO | GOBP_CELLULAR_KETONE_METABOLIC_PROCESS |
| GO | GOBP_CELLULAR_RESPONSE_TO_KETONE |
| GO | GOBP_KETONE_BIOSYNTHETIC_PROCESS |
| GO | GOBP_KETONE_BODY_BIOSYNTHETIC_PROCESS |
| GO | GOBP_KETONE_BODY_METABOLIC_PROCESS |
| GO | GOBP_KETONE_CATABOLIC_PROCESS |
| GO | GOBP_REGULATION_OF_CELLULAR_KETONE_METABOLIC_PROCESS |
| GO | GOBP_REGULATION_OF_KETONE_BIOSYNTHETIC_PROCESS |
| GO | GOBP_RESPONSE_TO_KETONE |
| HP | HP_HYPERKETONEMIA |
| REACTOME | REACTOME_KETONE_BODY_METABOLISM |
| REACTOME | REACTOME_SYNTHESIS_OF_KETONE_BODIES |
| WP | WP_DISORDERS_IN_KETONE_BODY_SYNTHESIS |

**Supplementary table 2:**

List of ketone-related genes

| AADAT | CLN3 | FMO4 | NDUFA9 | STARD3 | ELK1 |
| --- | --- | --- | --- | --- | --- |
| ABCB11 | CNR1 | FSHB | NMT1 | STARD4 | ERRFI1 |
| ABCD1 | COQ10A | GATD1 | NQO1 | STAT5B | FBP1 |
| ABCD2 | COQ10B | GHSR | NQO2 | SULT1C4 | FBXO32 |
| ACACB | COQ2 | GIP | NR1D1 | TDO2 | FDX1 |
| ACADL | COQ3 | GLO1 | NR1H2 | TPI1 | FOS |
| ACADVL | COQ4 | GOT2 | NR1H3 | TREX1 | GAS6 |
| ACMSD | COQ5 | GPD1 | NR1H4 | TRIB3 | GJB2 |
| ADH4 | COQ6 | GPD2 | NR4A3 | TWIST1 | GNAI1 |
| ADIPOQ | COQ7 | H6PD | OXCT1 | TYSND1 | GNB1 |
| ADM | COQ8A | HAGH | PANK2 | UBIAD1 | GNG2 |
| AFMID | COQ8B | HSD11B2 | PARK7 | UGT1A8 | GOLPH3 |
| AFP | COQ9 | HSD17B1 | PDK1 | VKORC1 | IGF1R |
| AIFM2 | CPT1A | HSD17B10 | PDK2 | VKORC1L1 | JAK2 |
| AKR1A1 | CRYZL1 | HSD17B3 | PDK3 | WDTC1 | KLF2 |
| AKR1B1 | CYP11A1 | HSD17B6 | PDK4 | WNT4 | KLF9 |
| AKR1B10 | CYP11B1 | IDO1 | PDSS1 | ACACA | LARP1 |
| AKR1C1 | CYP11B2 | IDO2 | PDSS2 | ACE | MAP4K1 |
| AKR1C2 | CYP17A1 | IL1B | PIBF1 | ACOD1 | METTL21C |
| AKR1C3 | CYP19A1 | INS | PLA2G3 | ADAM15 | MIR342 |
| AKR1C4 | CYP2B6 | INSIG1 | PLIN5 | ADCY1 | MSN |
| AKR7A2 | CYP46A1 | INSIG2 | PNKD | ADCY2 | MSTN |
| AKT1 | CYP4F11 | IRS1 | PPARA | ADCY3 | NCF1 |
| AKT2 | CYP4F12 | IRS2 | PPARD | ADCY5 | NCOA4 |
| ALDH8A1 | CYP4F2 | KDSR | PPARGC1A | ADCY6 | NDOR1 |
| ANXA1 | CYP7A1 | KLHL25 | PPTC7 | ADCY8 | NPAS4 |
| APOA4 | DAB2 | KMO | PRKAG2 | AHR | NR3C1 |
| APOA5 | DCAF5 | KYAT1 | PRKCE | AIFM1 | P2RY4 |
| APOC1 | DGAT2 | KYAT3 | PRMT3 | AKAP8 | P2RY6 |
| APOC2 | DGKQ | KYNU | PROX1 | AQP1 | PCK1 |
| APOC3 | DHRS4 | LHB | PTGS2 | AR | PCK2 |
| APPL2 | DHRS9 | LONP2 | RDH10 | ARG1 | PLAT |
| ATCAY | DKK3 | LPGAT1 | REST | ASS1 | POSTN |
| ATP2B4 | DKKL1 | MALRD1 | RGN | ATP2B1 | PRKAA1 |
| AVP | EDNRB | MFSD2A | SCAP | ATP5F1A | PRKAA2 |
| AVPR1A | EGR1 | MID1IP1 | SCNN1B | AXIN2 | PTGDR |
| BCKDK | EIF6 | MIR132 | SIRT1 | BMI1 | PTGER2 |
| BHMT | ELOVL5 | MIR182 | SIRT4 | CASP9 | PTGER4 |
| BMP2 | ERFE | MIR204 | SIRT5 | CDK4 | PTGFR |
| BMP5 | ERLIN1 | MIR21 | SIRT6 | CFLAR | RECQL5 |
| BMP6 | ERLIN2 | MIR30C1 | SLC22A13 | CFTR | ROCK2 |
| BRCA1 | ETFBKMT | MIR33A | SLC45A3 | CKB | RPS6KB1 |
| CACNA1H | FABP1 | MIR548P | SLC7A11 | CREB1 | RWDD1 |
| CAV1 | FABP3 | MIR766 | SLC7A7 | CYP1B1 | SCNN1A |
| CBR1 | FABP5 | MIR96 | SNCA | DCPS | SCNN1D |
| CBR4 | FDXR | MLXIPL | SOX9 | DDIT4 | SCNN1G |
| CD74 | FGF19 | MLYCD | SRD5A1 | DEFB104A | SERPINF1 |
| CEACAM1 | FGFR4 | MTLN | SRD5A2 | DEFB104B | SFRP1 |
| CES1 | FMO1 | NCOR1 | SREBF1 | EFNA5 | SGK1 |
| CLCN2 | FMO2 | NCOR2 | STAR | EIF4E | SLC39A9 |
| TAT | THBS1 | WBP2 | BDH1 | TGFB3 | TYMS |
| TBXA2R | TLR2 | YAP1 | BDH2 | TGFBR3 | UBE3A |
| TGFB1 | TSPO | TGFB2 | TXNIP | TH | UCN3 |
| DSG1 | SLIT3 | CLDN4 | RPL27 | CCND1 | PIK3CA |
| EDN1 | STK39 | CPS1 | SCN11A | CCR7 | POR |
| EPO | TACR1 | CSN1S1 | SIN3A | CD38 | PPP1R9B |
| PHKA2 | COX6B1 | CYBA | SLC12A3 | CDA | PSPH |
| PHKG2 | GCDH | CYBB | SLIT2 | CLDN1 | PTAFR |
| BAD | NEFL | HMGCS2 | HSD3B1 | UCP1 | GPI |
| BCL2L1 | NKX2.2 | SLC27A5 | HSD3B2 | UGT3A2 | HDAC6 |
| BGLAP | NKX3.1 | ACAT1 | HSF1 | USP8 | HNRNPD |
| CA9 | NTRK3 | OXCT2 | IGFBP7 | VPS54 | HOXA11 |
| CALM3 | OXT | TYRP1 | MAOB | XRN1 | HOXA13 |
| CALR | OXTR | A2M | MBP | AACS | HOXA9 |
| CBL | PAPPA | AANAT | MTAP | ACSS3 | HOXB13 |
| CCL19 | PARP1 | ABCB4 | NASP | HMGCL | HOXD13 |
| CCL21 | PCNA | ABHD2 | NCOA2 | HMGCLL1 | HPCA |
| SLC5A5 | F5 | SPHK2 | FOSL1 | TNFSF4 | GBA1 |
| SMYD3 | F7 | SPP1 | FOXP1 | TRERF1 | GLB1 |
| SOX10 | FIBIN | SRC | FOXP3 | SLC37A4 | GABRB1 |
| SP1 | FOSB | TFAP4 |  |  |  |

**Supplementary table 3:**

List of ferroptosis-related genes

| RPL8 | BAP1 | SLC7A5 | NGB | CA9 | DDIT3 |
| --- | --- | --- | --- | --- | --- |
| IREB2 | ABCC1 | HERPUD1 | YWHAE | TMBIM4 | JDP2 |
| ATP5MC3 | MIR6852 | XBP1 | GABPB1 | PLIN2 | SESN2 |
| CS | ACVR1B | SLC3A2 | AURKA | AIFM2 | SLC1A4 |
| EMC2 | TGFBR1 | CBS | MIR4715 | LAMP2 | PCK2 |
| ACSF2 | IFNG | ATF4 | RIPK1 | ZFP36 | TXNIP |
| NOX1 | ANO6 | ZNF419 | PRDX1 | PROM2 | VLDLR |
| CYBB | HMGB1 | KLHL24 | MIR30B | CHMP5 | GPT2 |
| NOX3 | TNFAIP3 | TRIB3 | GPX4 | CHMP6 | PSAT1 |
| NOX4 | ATF3 | ZFP69B | AKR1C1 | CAV1 | LURAP1L |
| NOX5 | ATM | ATP6V1G2 | AKR1C2 | GCH1 | SELENOS |
| DUOX1 | YY1AP1 | VEGFA | AKR1C3 | GCLC | ANGPTL7 |
| DUOX2 | EGLN2 | GDF15 | RB1 | FANCD2 | SLC7A11 |
| G6PD | MIOX | TUBE1 | HSPB1 | FTMT | DDIT4 |
| PGD | TAZ | ARRDC3 | HSF1 | MTOR | LOC284561 |
| VDAC2 | MTDH | CEBPG | NFE2L2 | ENPP2 | ASNS |
| TP53 | IDH1 | SNORA16A | SQSTM1 | MIR212 | TSC22D3 |
| ACSL4 | FBXW7 | RGS4 | NQO1 | Fer1HCH | TXNRD1 |
| LPCAT3 | PANX1 | BLOC1S5-TXNDC5 | MUC1 | CDKN2A | SRXN1 |
| NRAS | DNAJB6 | LOC390705 | MT1G | PEBP1 | GPX2 |
| KRAS | LONP1 | EIF2S1 | SLC40A1 | SOCS1 | BNIP3 |
| HRAS | PTGS2 | HSD17B11 | CISD1 | CDO1 | OXSR1 |
| CARS1 | DUSP1 | AGPAT3 | HSPA5 | MYB | UBC |
| KEAP1 | NOS2 | SETD1B | HELLS | CHAC1 | ALB |
| HMOX1 | NCF2 | TF | SCD | LINC00472 | PRKAA1 |
| ATG5 | MT3 | FTL | FADS2 | SAT1 | MAPK1 |
| ATG7 | ALOX12 | ALOX15 | ALOXE3 | EGFR | ZEB1 |
| EIF2AK4 | OTUB1 | HBA1 | LINC00336 | PLIN4 | PRDX6 |
| ALOX5 | CD44 | NNMT | BRD4 | HIC1 | MIR17 |
| STMN1 | NF2 | CAPG | HIF1A | SLC2A1 | FH |
| RRM2 | ARNTL | HNF4A | JUN | SLC2A3 | CISD2 |
| SLC2A6 | MIR9-1 | SLC2A12 | MIR9-3 | SLC2A14 | ACSL3 |
| SLC2A8 | MIR9-2 | GLUT13 | ISCU | FTH1 | PML |
| DRD5 | NFS1 | MAP3K5 | CDKN1A | TFRC | SRC |
| DRD4 | TP63 | MAPK14 | MIR137 | MAFG | STAT3 |
| PRKAA2 | ELAVL1 | MAPK3 | DPP4 | ALOX15B | PHKG2 |

**Supplementary figure legends**

**Supplementary figure 1:** Flow chart of this study.

**Supplementary figure 2:** Sample clustering diagram of WGCNA.

**Supplementary figure 3:** (A) Selection of the optimal soft thresholding power β. (B) KEGG enrichment (left) and GO enrichment (right) of ferroptosis-stage DEGs.

**Supplementary figure 4:** (A) Forest plot of univariate Cox regression of ferroptosis-stage DEGs. (B) Kaplan–Meier curves of ketone-stage-survival DEGs. (C) Kaplan-Meier curves of ferroptosis-stage-survival DEGs. *P* < 0.1.

**Supplementary figure 5:** (A) 22 Immune cell infiltration for all samples. (B) Different immune cell infiltration between normal and tumor tissues. (C) Subnetwork of genes with the highest score in Figure 3F with their first nodes.

**Supplementary figure 6:** (A) Violin plot of the ketogenic phenotype among cell types. (B) The violin plot of the ferroptosis phenotype among cell types.

**Supplementary figure 7:** (A) PANC1 was treated with a gradient concentration of Na-OHB, and dead cells were stained with SYTOX Orange; the histogram is shown in Figure 5C. (B) Cells were treated with Na-OHB with or without Fer-1, and dead cells were stained with SYTOX Orange; the histogram is shown in Figure 5E. (C) IP of SLC7A11 in PANC1 cells and ubiquitin was detected. (D) Pan02 cells were treated with Na-OHB with or without Fer-1, and dead cells were stained with SYTOX Orange. (E) Intracellular Fe^2+^ levels within Pan02 cells were assessed using flow cytometry. (F) Lipid ROS levels within Pan02 cells were measured by flow cytometry. (G) GSH/GSSG ratio of Pan02 cell after being treated by Na-OHB/Fer-1. (H) Intracellular cystine was assessed using flow cytometry. (I) Western blotting of proteins in AMPK/NRF2 pathway after being treated by Na-OHB in Pan02. (J) Western blotting of CAV1, SLC7A11, and α-tubulin after being treated by Na-OHB with or without MG-132 in Pan02. **D-H,** data are shown as the mean ± S.E.M., *n* = 3, two-tailed *t* test or one-way ANOVA with *Dunnett-t* test. **I** and **J,** data are shown as the mean ± SD, *n* = 3, one-way ANOVA with *Dunnett-t* test. ns (not significant), * *P* < 0.05, ** *P* < 0.01, and *** *P* < 0.001.

**Supplementary figure 8:** (A) Western blotting of CAV1, SLC7A11, and α-Tubulin in MIA PaCa-2, MIA PaCa-2^-NC^, and MIA PaCa-2^-OE^ cell lines. (B) Western blotting of CAV1, SLC7A11, and α-Tubulin in MIA PaCa-2, MIA PaCa-2^-shNC^, and MIA PaCa-2^-shCAV1^ cell lines. (C) Western blotting of CAV1, SLC7A11, and α-Tubulin in PANC1, PANC1^-NC^, and PANC1^-OE^ cell lines. (D) Western blotting of CAV1, SLC7A11, and α-Tubulin in PANC1, PANC1^-shNC^, and PANC1^-shCAV1^ cell lines. (E) qPCR of *SLC7A11* for MIA PaCa-2, MIA PaCa-2^-OE^, MIA PaCa-2^-shCAV1^ cell lines (left) and for PANC1, PANC1^-OE^, and PANC1^-shCAV1^ cell lines. (F) Cell death ratio of MIA PaCa-2, MIA PaCa-2^-OE^, and MIA PaCa-2^-shCAV1^ cell lines treated with 0, 0.5, 1, 2, 4μM RSL3 (left) and cell death ratio of PANC1, PANC1^-OE^, and PANC1^-shCAV1^ cell lines treated with 0, 0.5, 1, 2, 4μM RSL3 (right). (G) Lipid ROS for MIA PaCa-2, MIA PaCa-2^-OE^, MIA PaCa-2^-shCAV1^ cell lines (left) and for PANC1, PANC1^-OE^, and PANC1^-shCAV1^ cell lines. (H) Mitochondrial membrane potential for MIA PaCa-2, MIA PaCa-2^-OE^, MIA PaCa-2^-shCAV1^ cell lines (left) and for PANC1, PANC1^-OE^, and PANC1^-shCAV1^ cell lines. (I) Mitochondrial mass for MIA PaCa-2, MIA PaCa-2^-OE^, MIA PaCa-2^-shCAV1^ cell lines (left) and for PANC1, PANC1^-OE^, and PANC1^-shCAV1^ cell lines. **A-D,** data are shown as the mean ± SD, *n* = 3, one-way ANOVA with *Dunnett-t* test. **E-H,** data are shown as the mean ± S.E.M., *n* = 3, one-way ANOVA with *Dunnett-t* test. ns (not significant), * *P* < 0.05, ** *P* < 0.01, *** *P* < 0.001, and **** *P* < 0.0001.

**Supplementary figure 9:** (A) Blood ketone (left) and glucose (right) levels of normal or ketogenic diet mice bearing Pan02 tumor. (B) Tumor weight and volume of normal or ketogenic diet mice bearing Pan02 tumor. (C) Representative image of multiplex immunofluorescence staining on Pan02 tumor issue sections fed with a normal diet. (D) Representative image of multiplex immunofluorescence staining on Pan02 tumor tissue sections fed with a ketogenic diet. (E) Percentage of CAV1^+^ (left) or SLC7A11^+^ (right) cells per field on Pan02 tumor tissue sections. (F) Percentage of gradient CAV1^+^ (left) or SLC7A11^+^ (right) cells on Pan02 tumor tissue sections. (G) Correlation scatter plot of MFI between CAV1 and SLC7A11 on Pan02 tumor tissue sections. (H) Representative image of multiplex immunofluorescence staining for 8-OHdG (green) and 4-HNE (red) on Pan02 tumor tissue sections fed with a normal diet. (I) Representative image of multiplex immunofluorescence staining for 8-OHdG (green) and 4-HNE (red) on Pan02 tumor tissue sections fed with a ketogenic diet. (J) Percentage of 4-HNE^+^ (left) or 8-OHdG^+^ (right) cells per field on Pan02 tumor tissue sections. **A, B, E, F,** and **J**, data are shown as the mean ± SD, *n* = 6, two-tailed *t* test or two-way ANOVA. **I,** spearman correlation analysis. ns (not significant), ** *P* < 0.01, *** *P* < 0.001, and **** *P* < 0.0001.
