# Supplementary figures and images for: Integrative insights into the role of CAV1 in ketogenic diet and ferroptosis in pancreatic cancer
Source: Cell Death Discov. 2025 Apr 4;11:139. doi: 10.1038/s41420-025-02421-z (PMC11968908; doi:10.1038/s41420-025-02421-z)

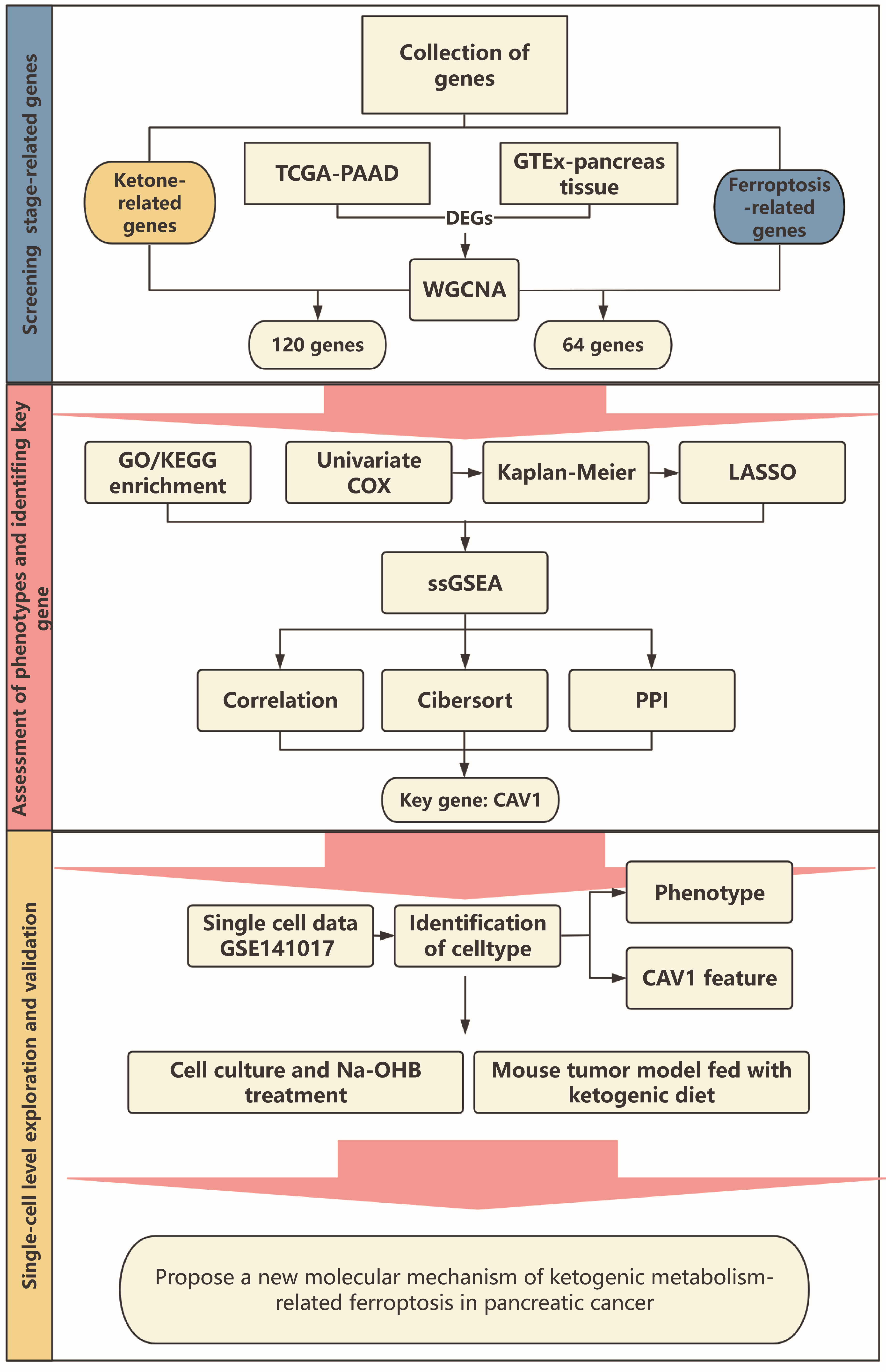

Supplement: Supplementary file 2 — Supplementary figure 1: Flow chart of this study. [file 41420_2025_2421_MOESM2_ESM.tif]

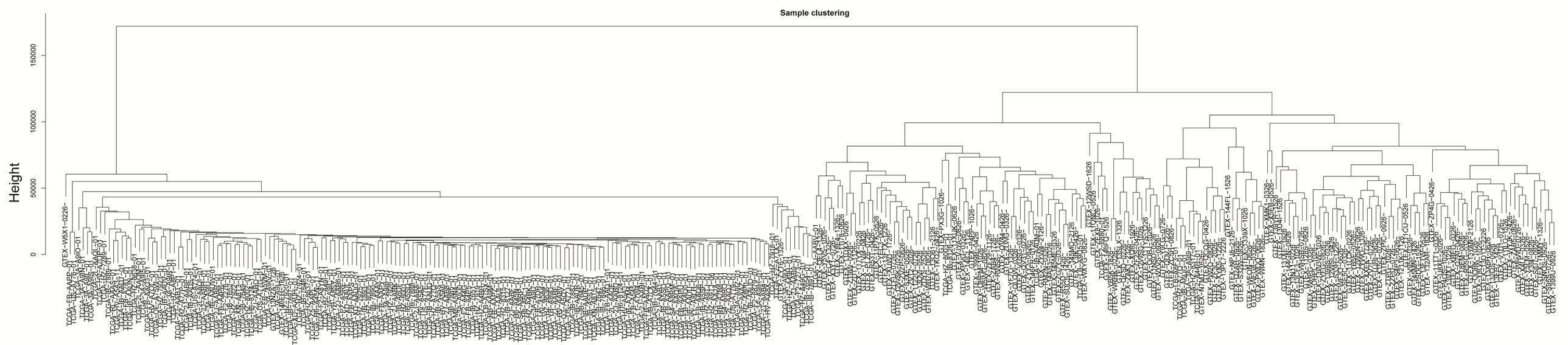

Supplement: Supplementary file 3 — Supplementary figure 2: Sample clustering diagram of WGCNA. [file 41420_2025_2421_MOESM3_ESM.tif]

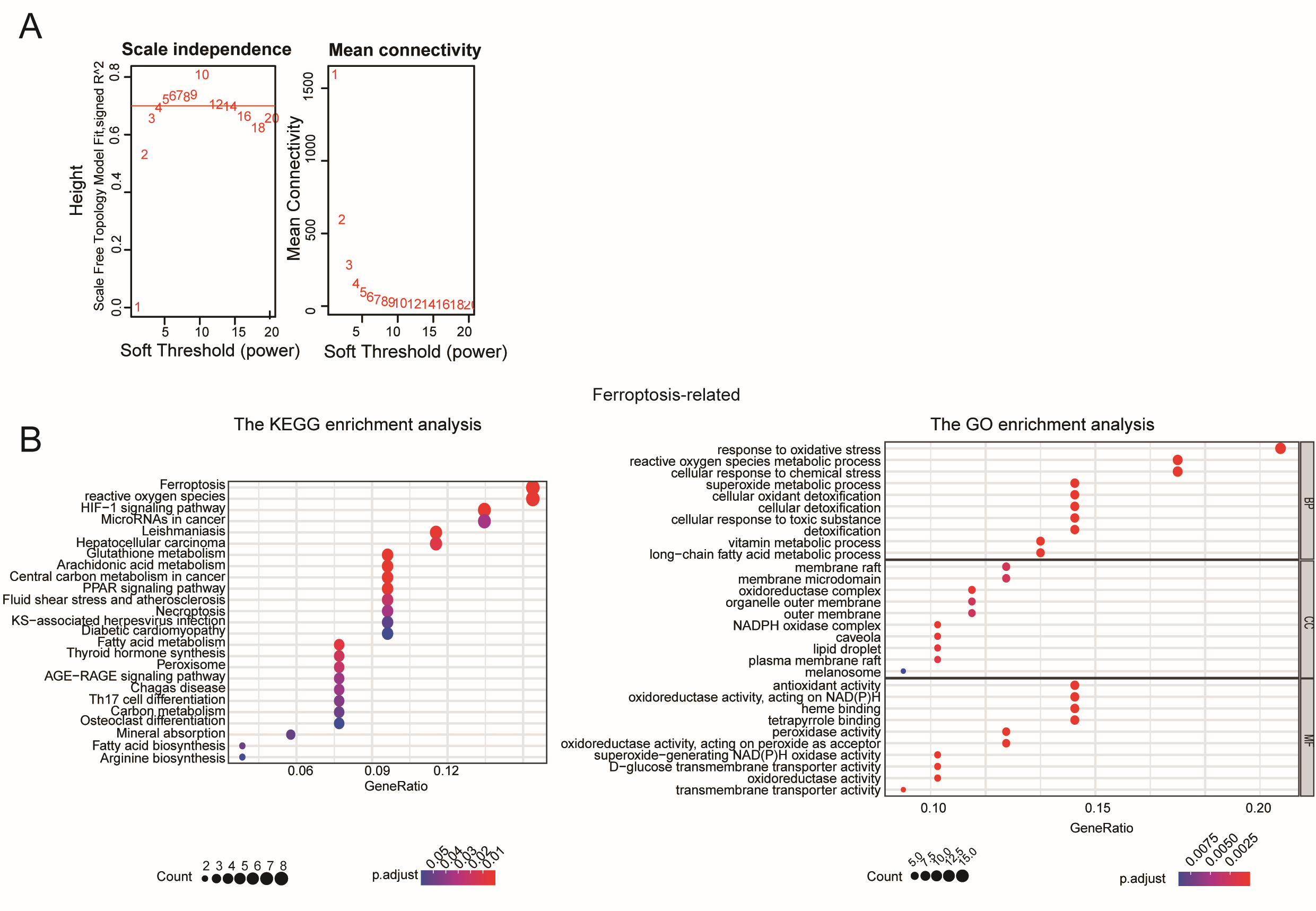

Supplement: Supplementary file 4 — Supplementary figure 3 [file 41420_2025_2421_MOESM4_ESM.tif]

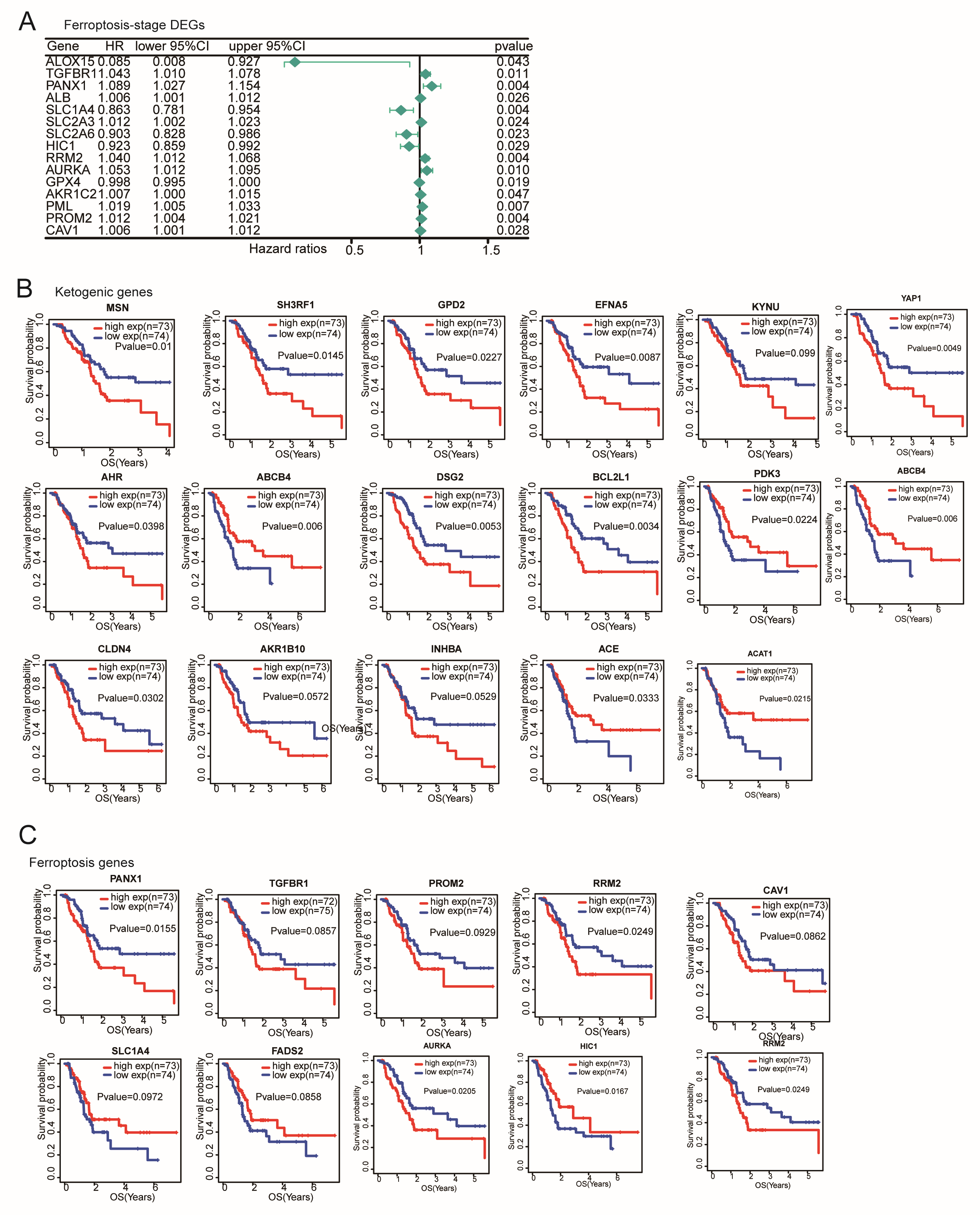

Supplement: Supplementary file 5 — Supplementary figure 4 [file 41420_2025_2421_MOESM5_ESM.tif]

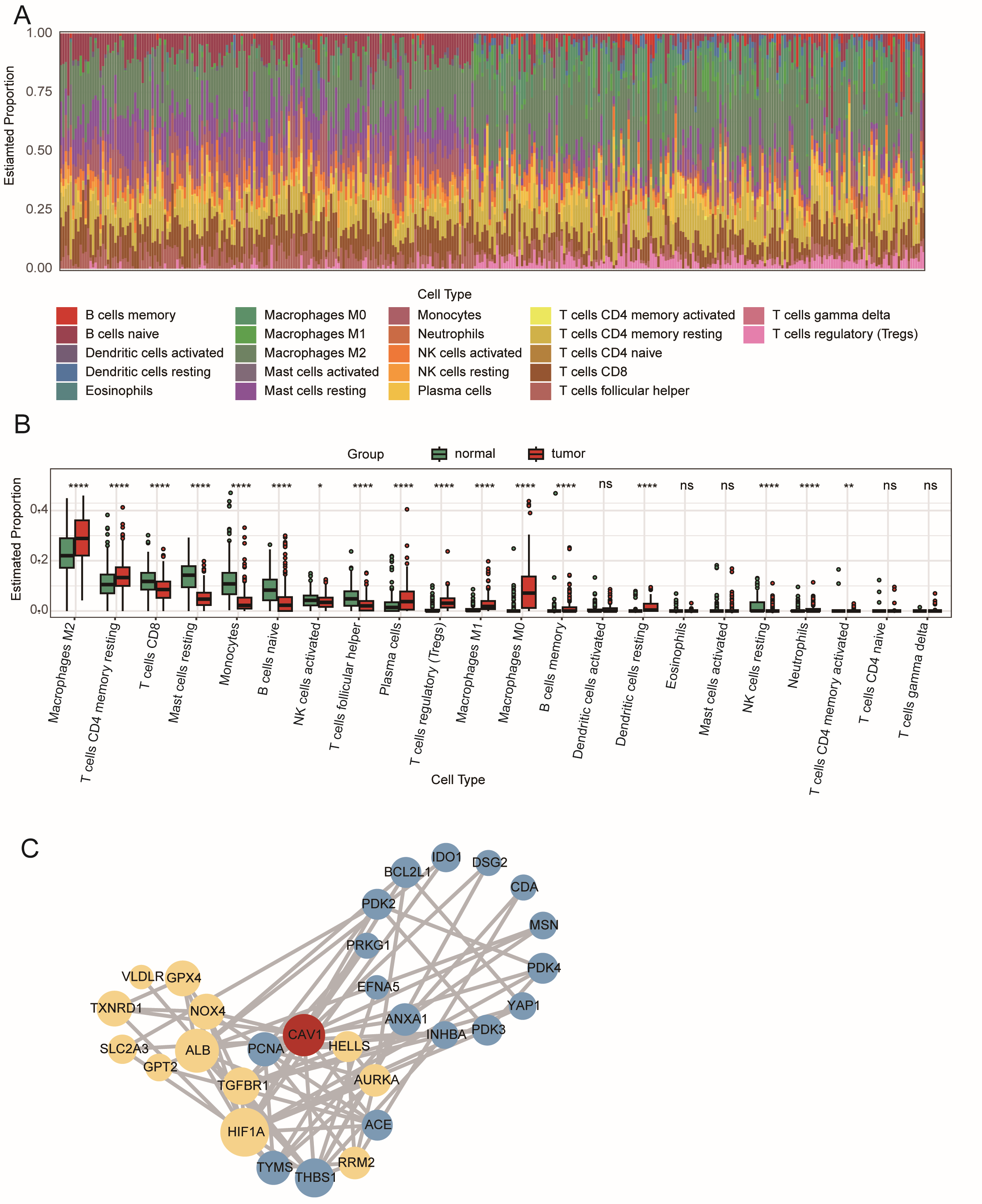

Supplement: Supplementary file 6 — Supplementary figure 5 [file 41420_2025_2421_MOESM6_ESM.tif]

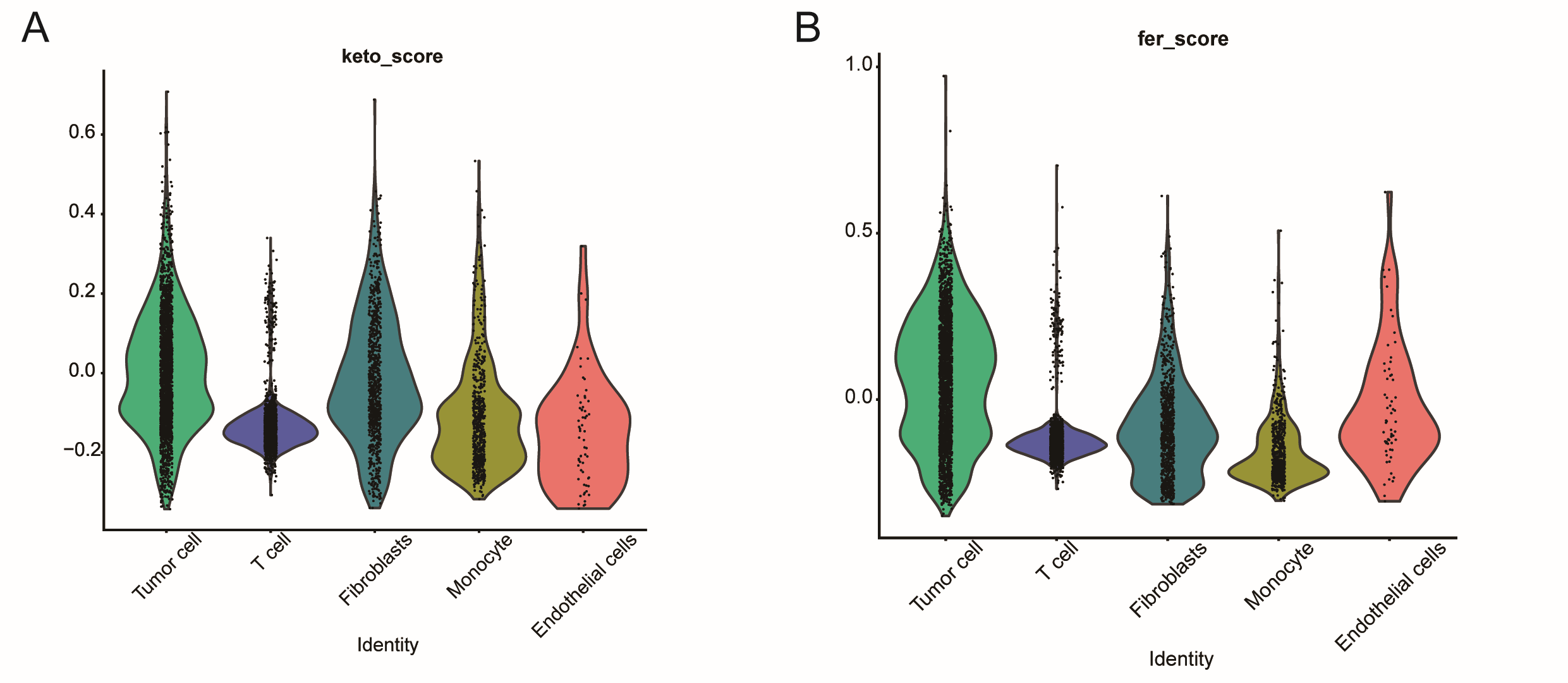

Supplement: Supplementary file 7 — Supplementary figure 6 [file 41420_2025_2421_MOESM7_ESM.tif]

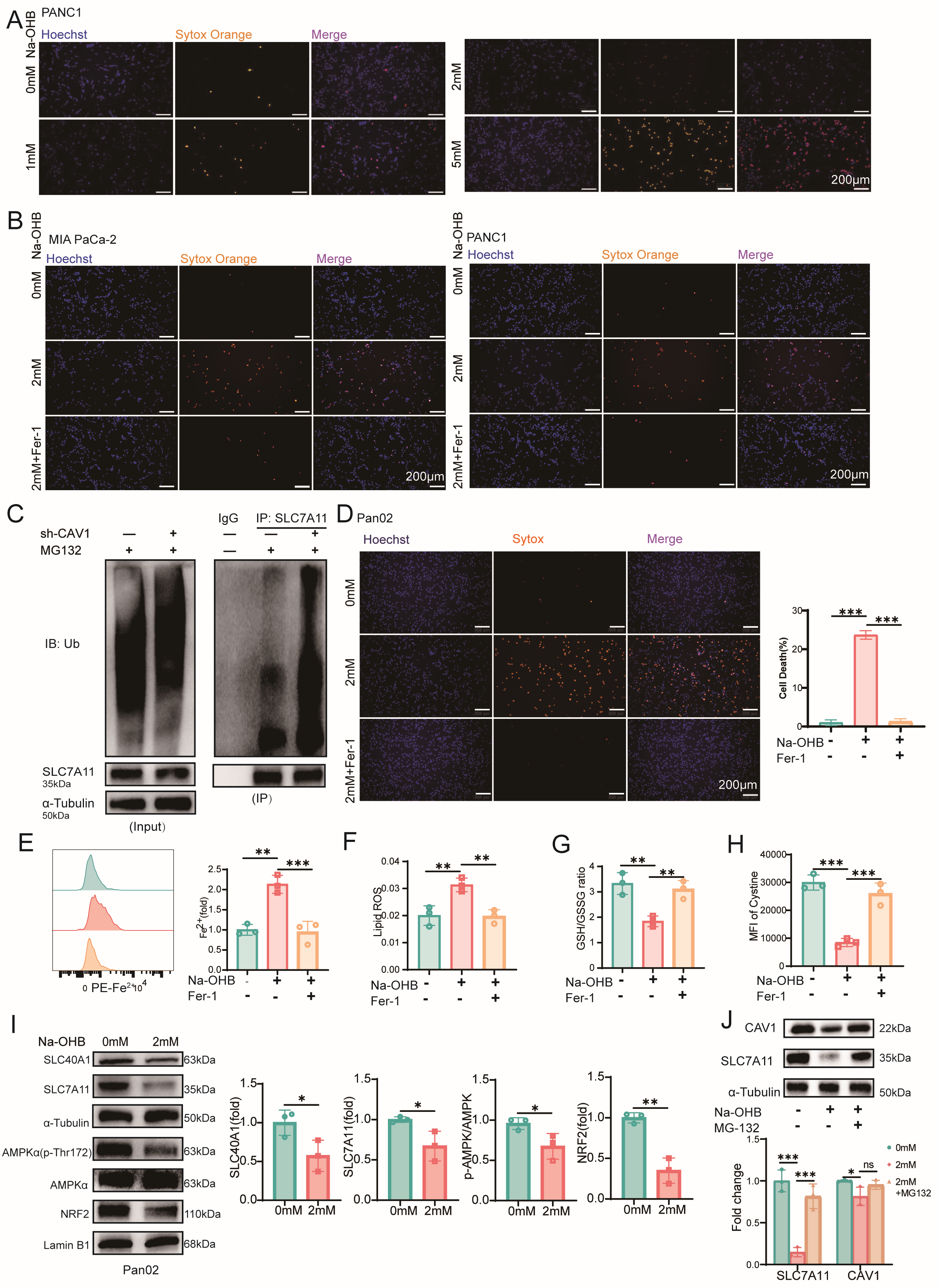

Supplement: Supplementary file 8 — Supplementary figure 7 [file 41420_2025_2421_MOESM8_ESM.tif]

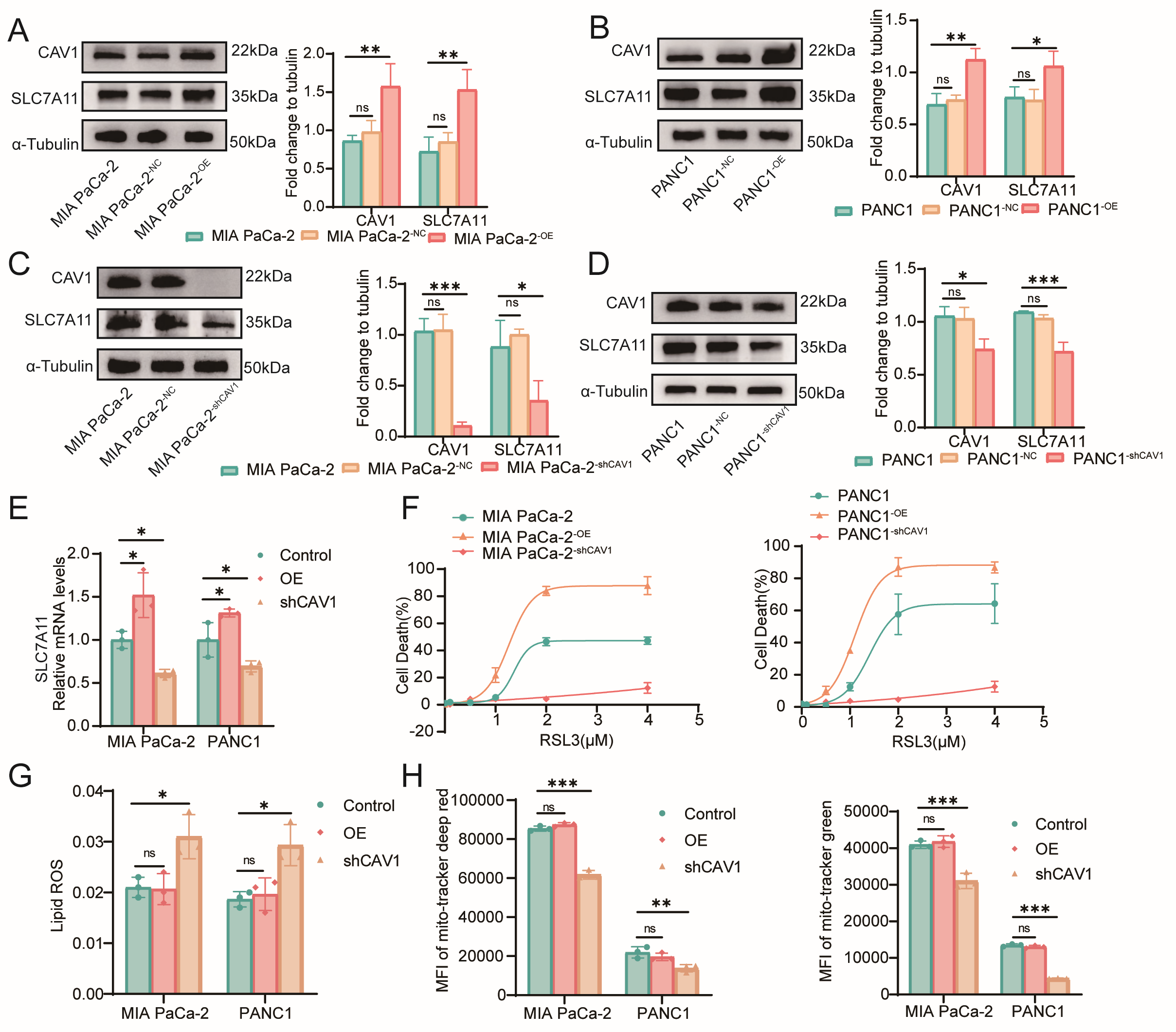

Supplement: Supplementary file 9 — Supplementary figure 8 [file 41420_2025_2421_MOESM9_ESM.tif]

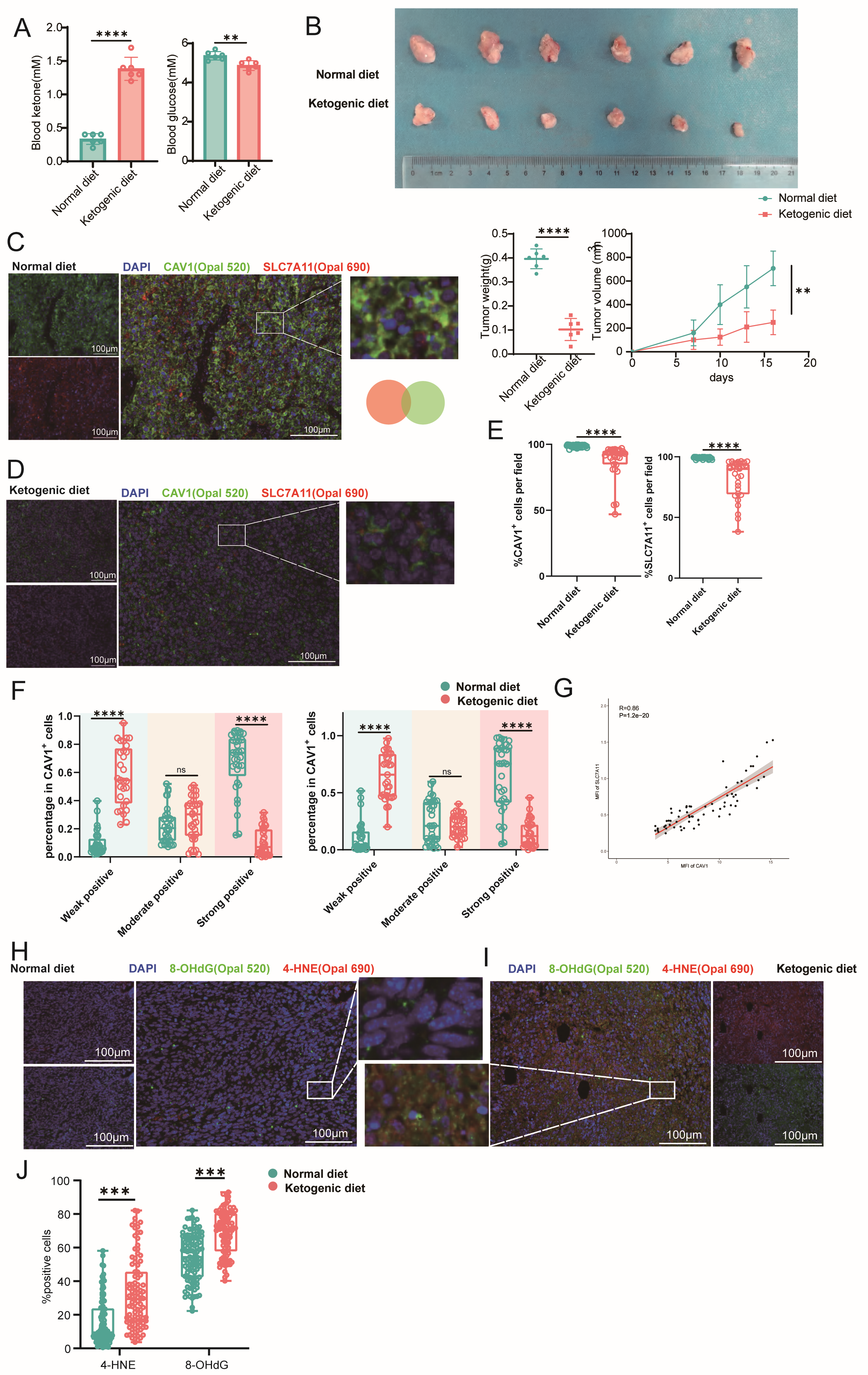

Supplement: Supplementary file 10 — Supplementary figure 9 [file 41420_2025_2421_MOESM10_ESM.tif]
