# Supplementary material for: Integrative insights into the role of CAV1 in ketogenic diet and ferroptosis in pancreatic cancer
Source: Cell Death Discov. 2025 Apr 4;11:139. doi: 10.1038/s41420-025-02421-z (PMC11968908; doi:10.1038/s41420-025-02421-z)
Supplement: Supplementary file 11 — Western blotting [file 41420_2025_2421_MOESM11_ESM.pdf]

Figure5A-CAV1

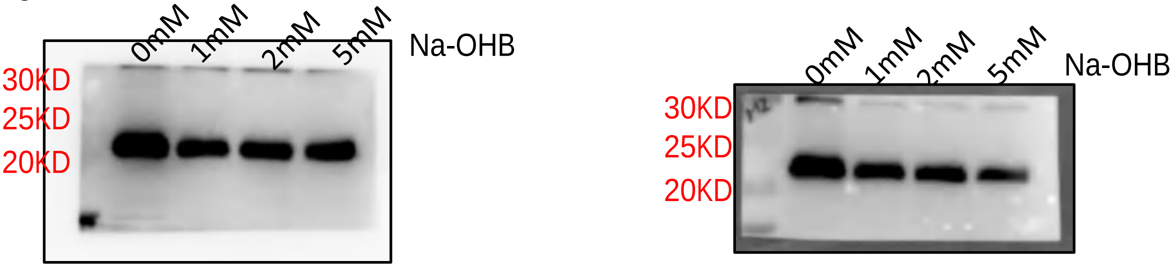

Figure5A-Tubulin

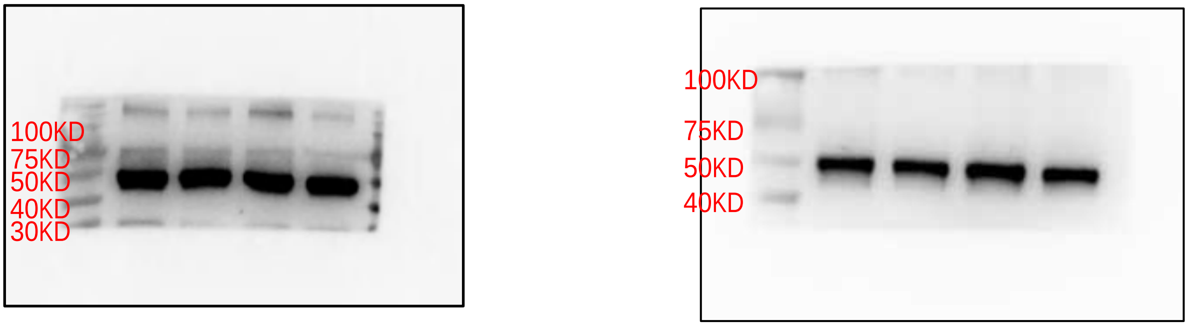

Figure6A-SLC40A1

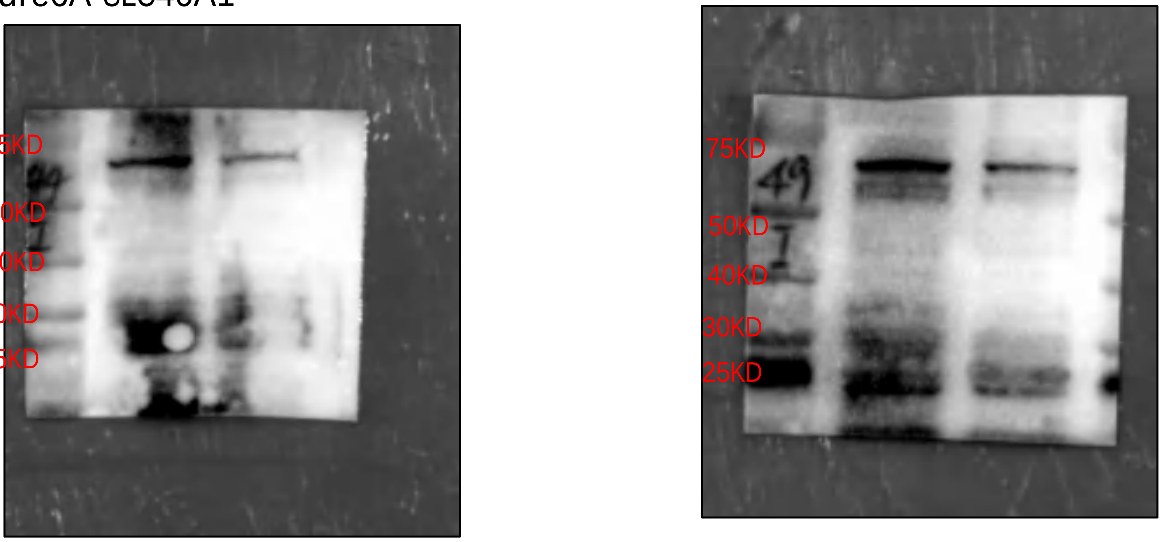

Figure6A-SLC7A11

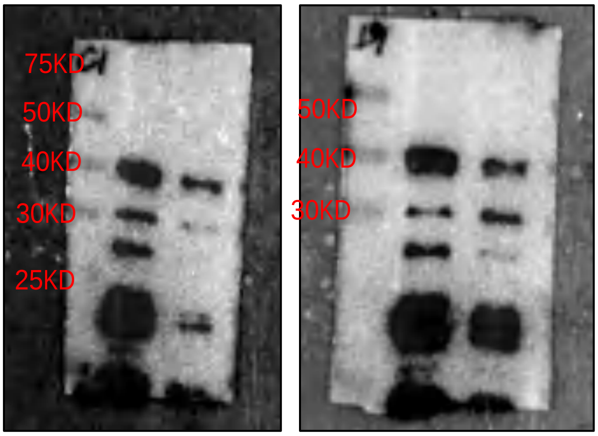

Figure6A-tubulin

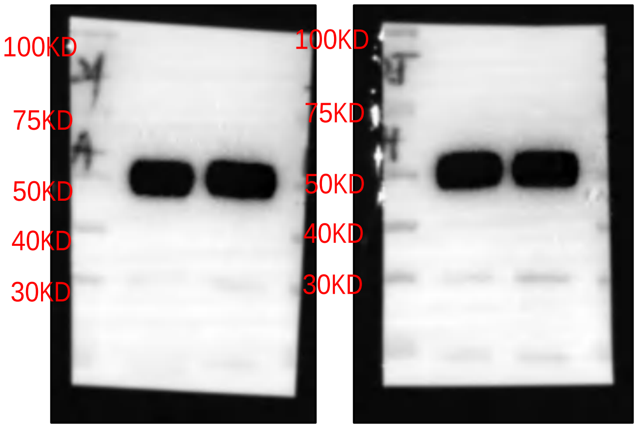

Figure6A-AMPK（p-Thr172）

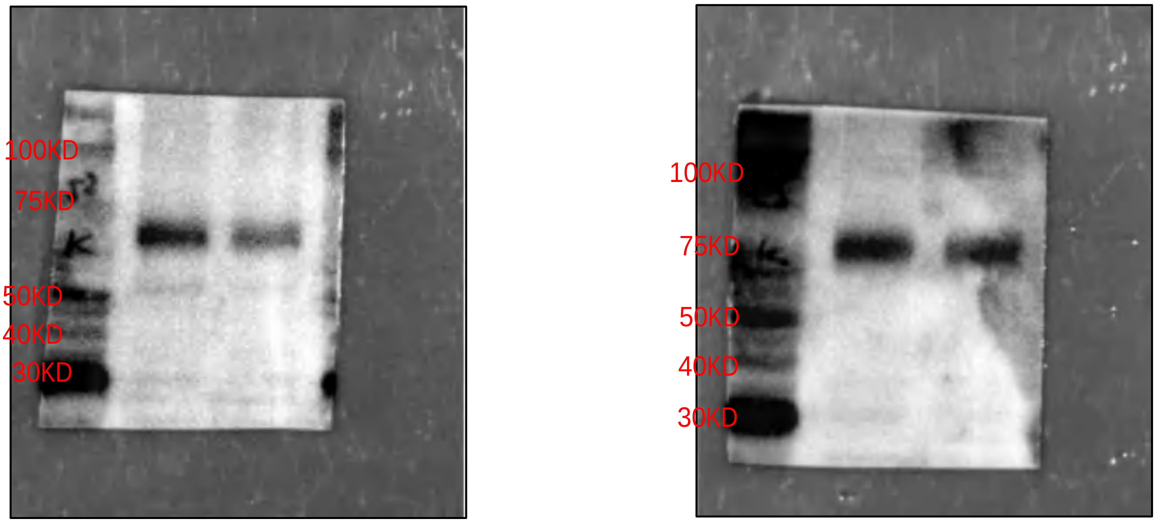

Figure6A-AMPK

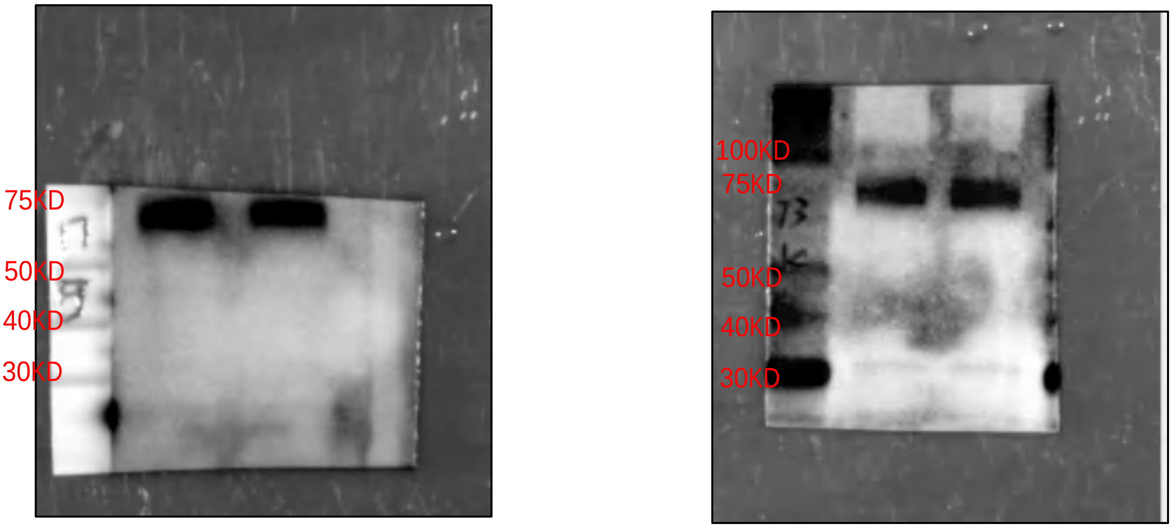

Figure6A-NRF2

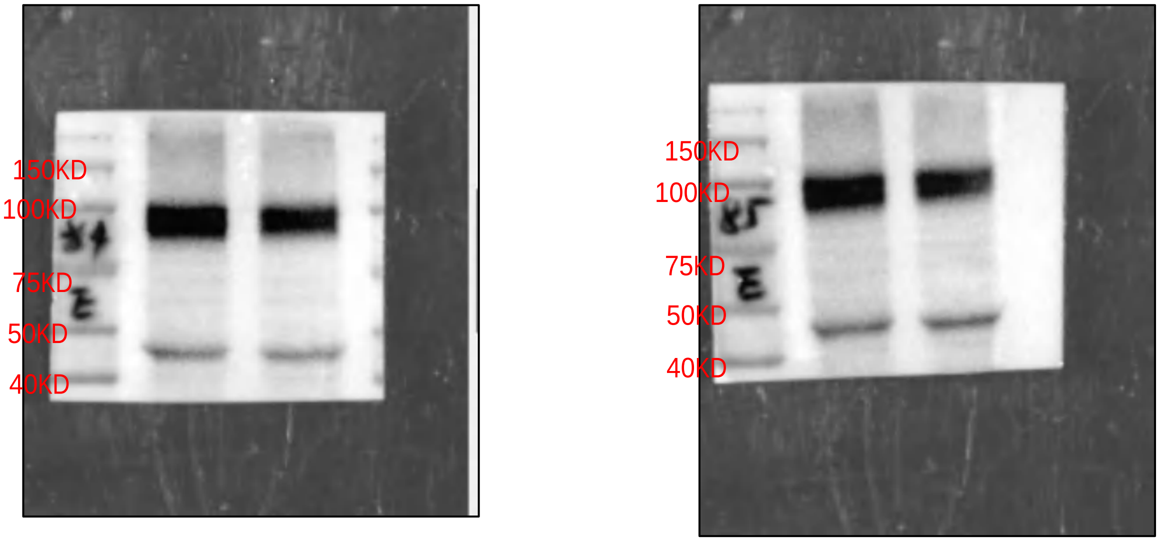

Figure6A-Lamin B1

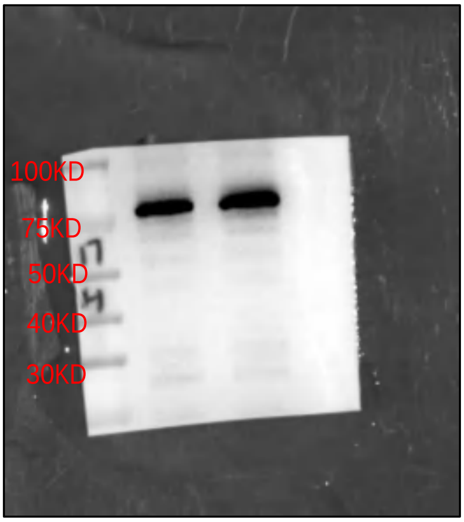

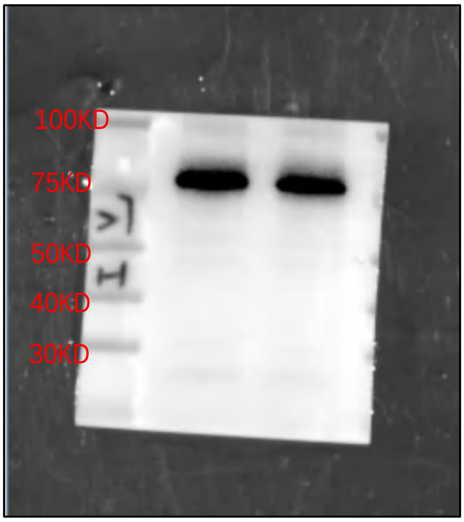

Figure6B-CAV1

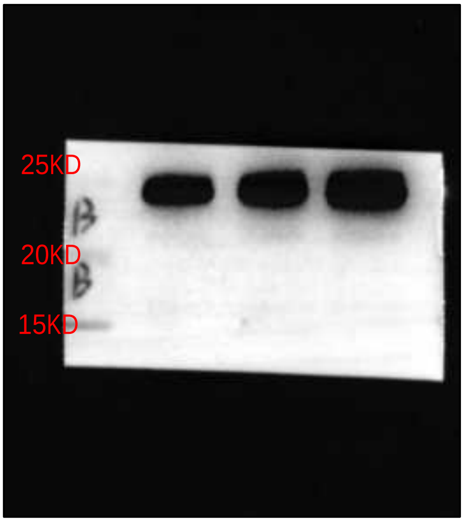

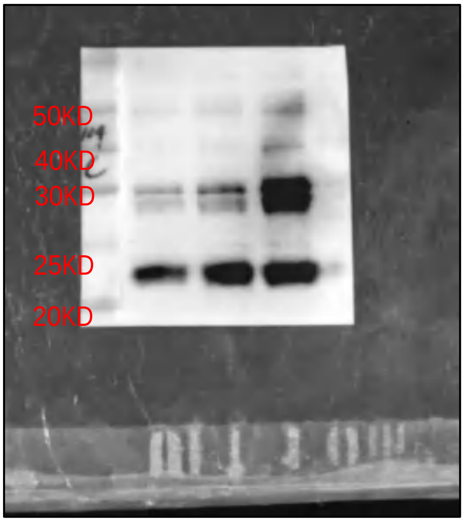

Figure6B-SLC40A1

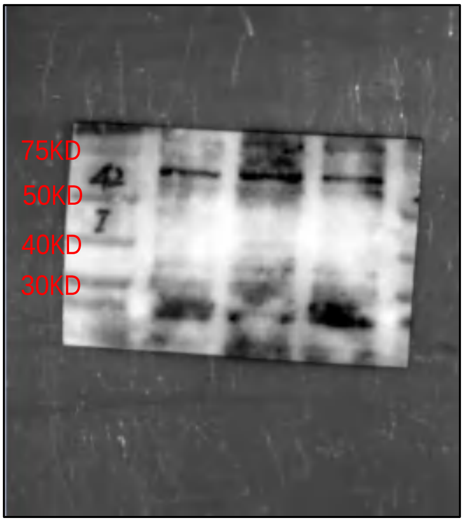

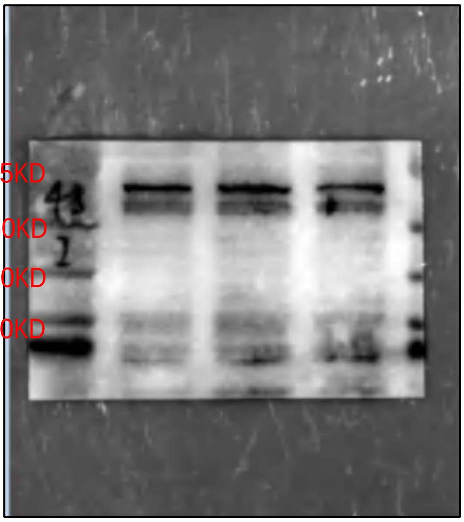

Figure6B-SLC7A11

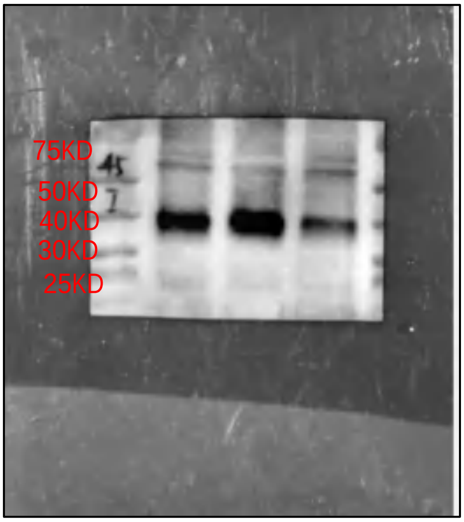

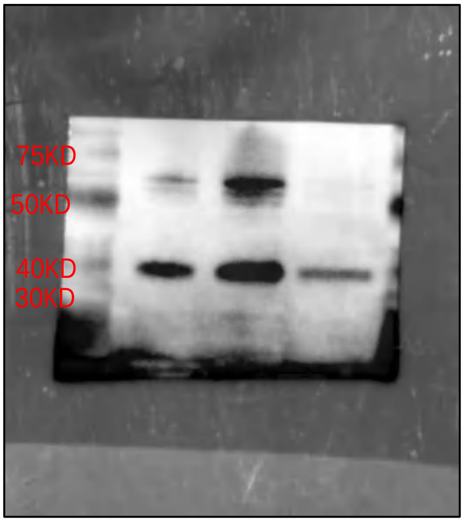

Figure6B-Tubulin

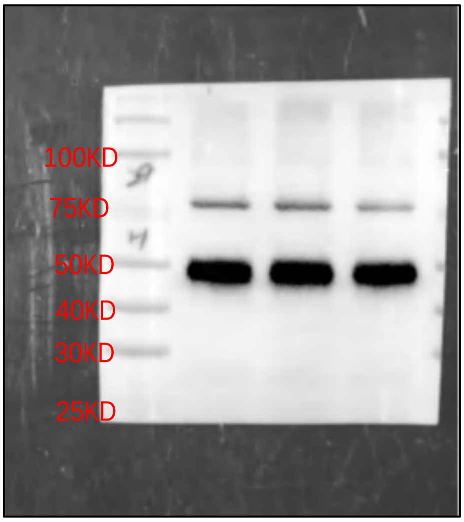

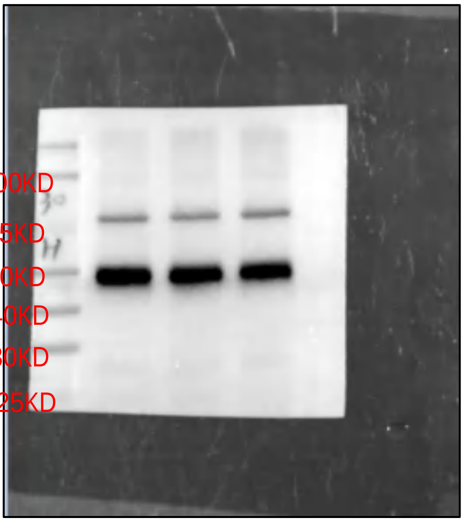

Figure6B-AMPK(p-Thr172)

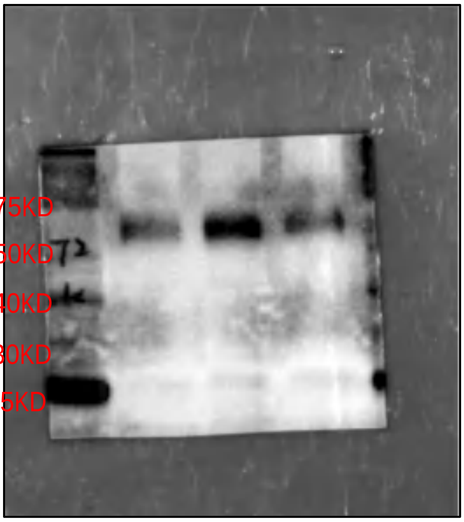

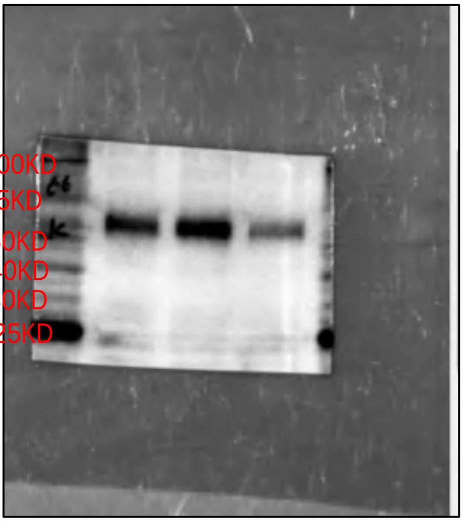

Figure6B-AMPK

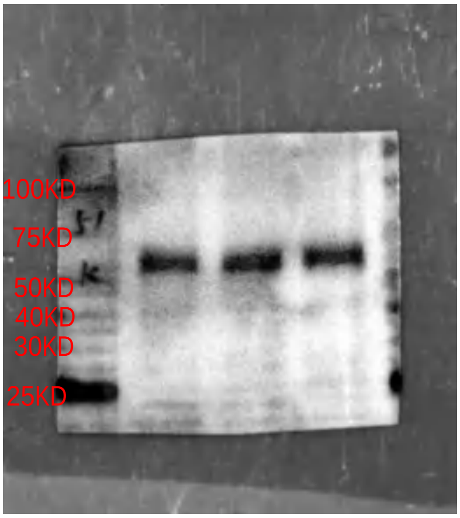

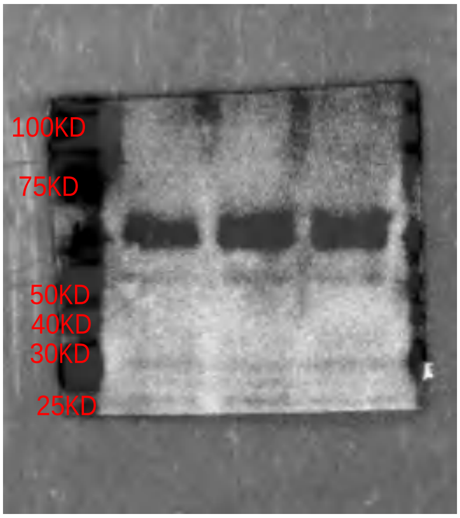

Figure6B-NRF2

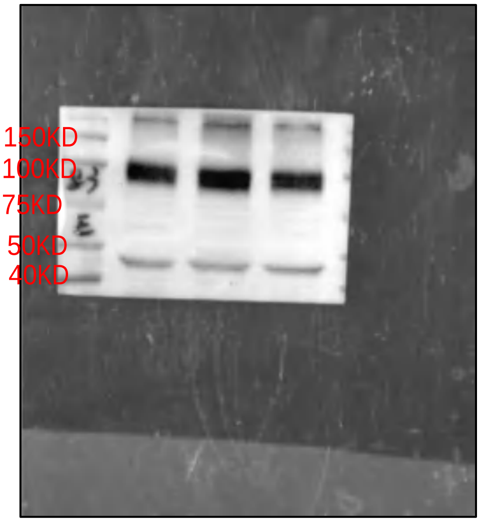

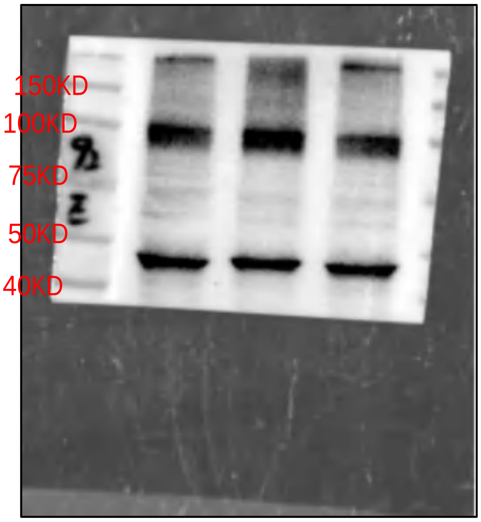

Figure6B-Lamin B1

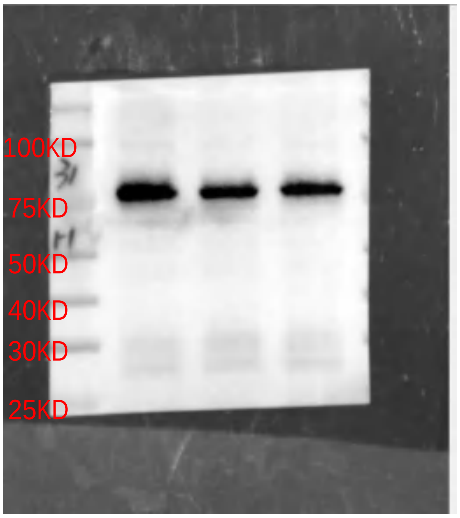

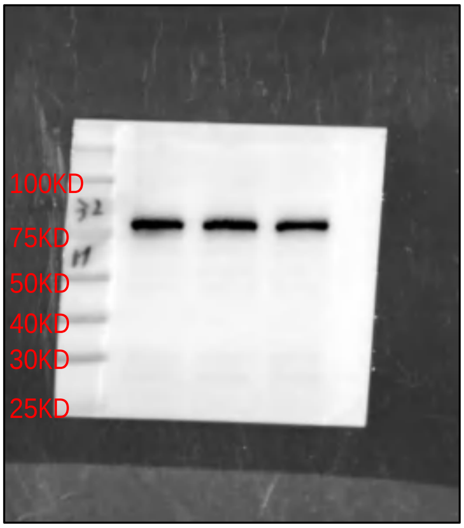

Figure6F-CAV1

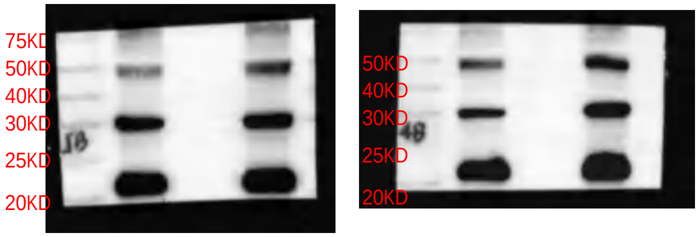

Figure6F-SLC7A11

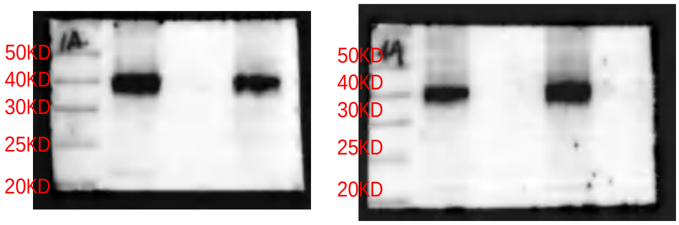

Figure6F-SLC40A1

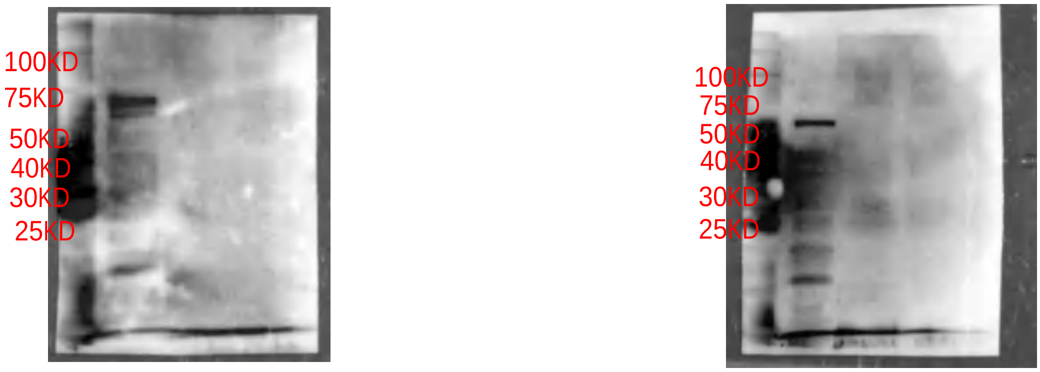

Figure6G-CAV1

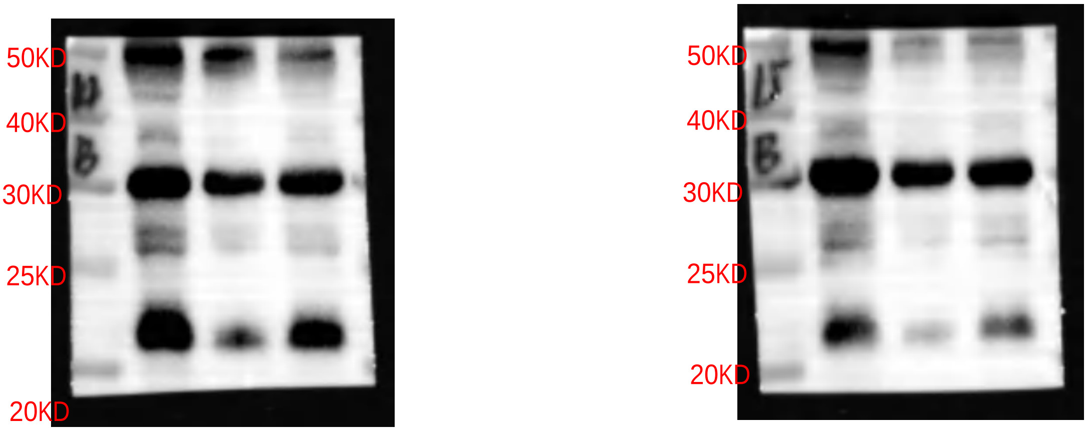

Figure6G-SLC7A11

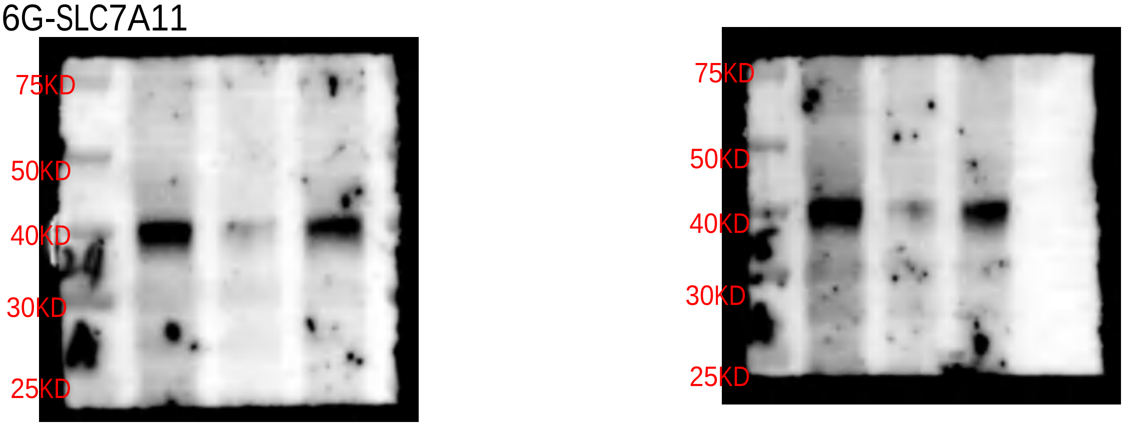

Figure6G-tubulin

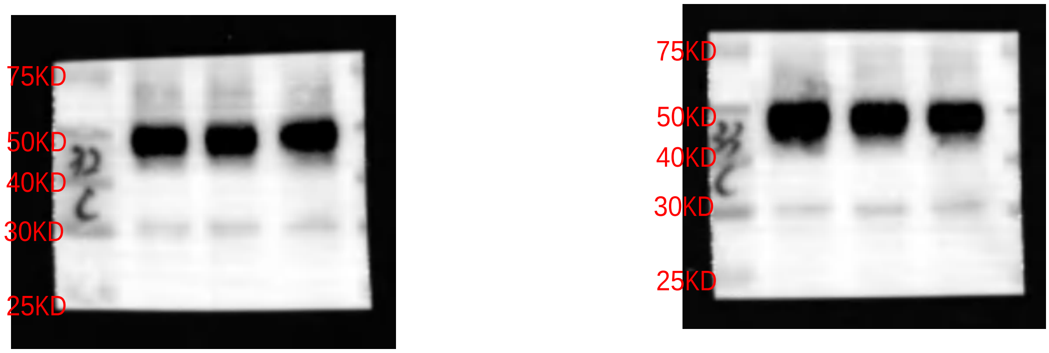

Figure6I-SLC7A11

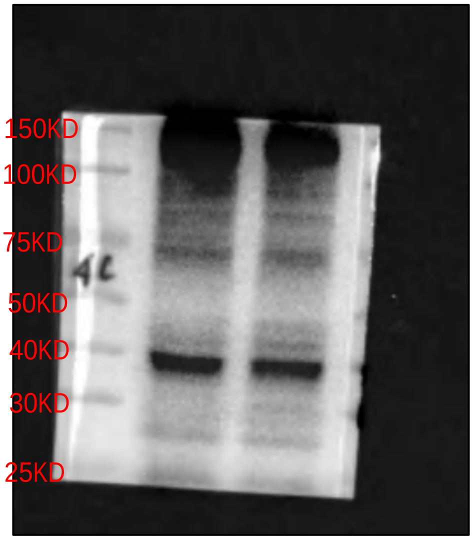

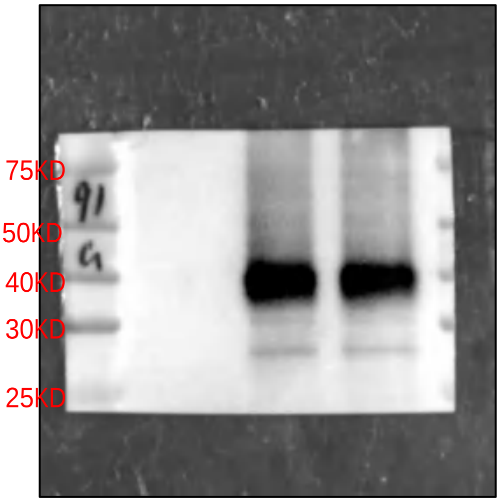

Figure6I-Ub

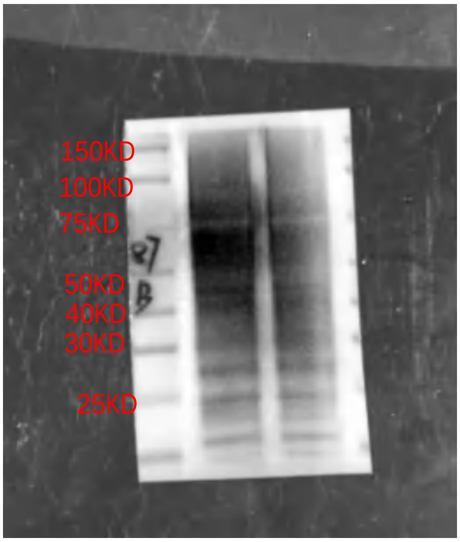

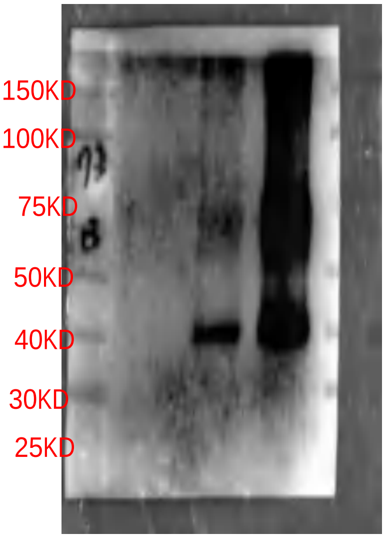

Figure6I-Tubulin

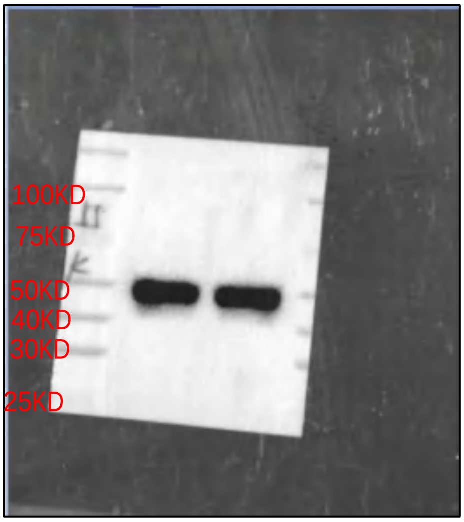

sFigure7C-Ub

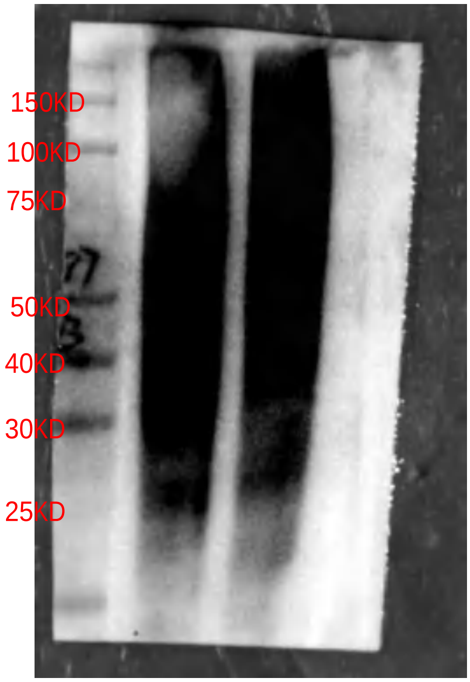

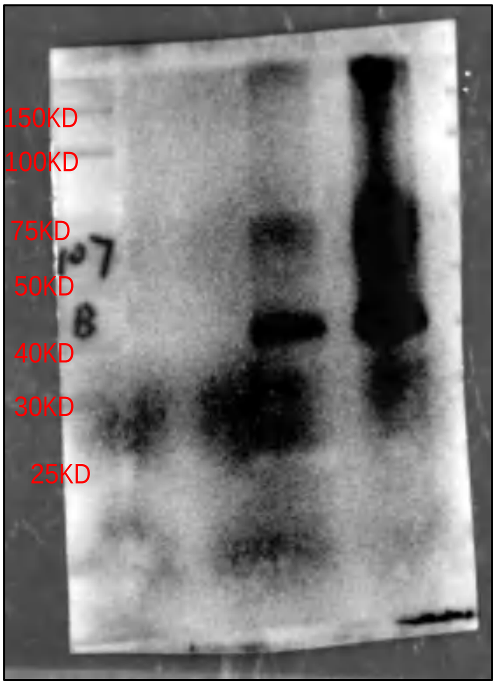

sFigure7C-SLC7A11

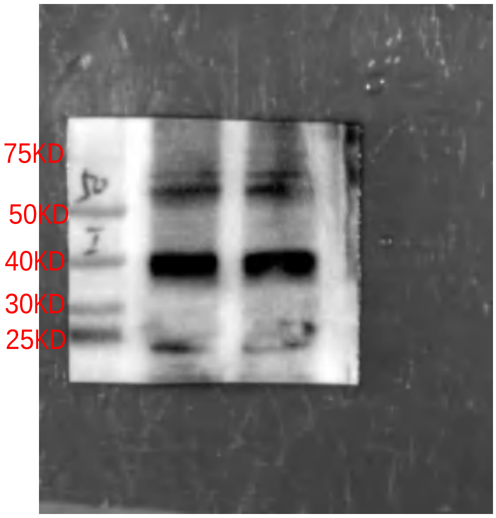

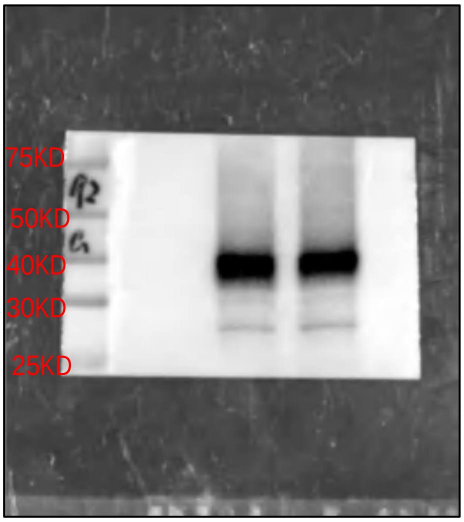

sFigure7C-Tubulin

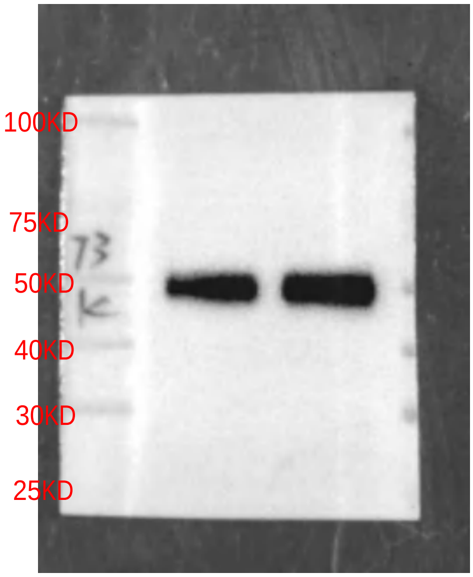

sFigure7I-SLC40A1

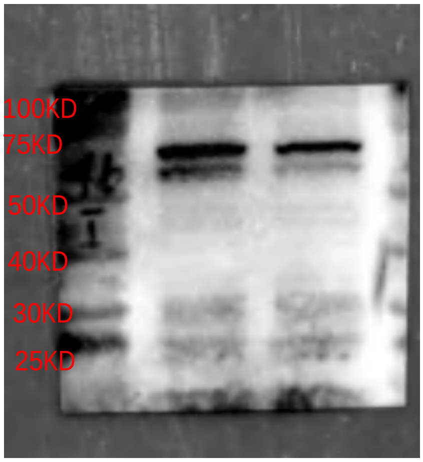

sFigure7I-SLC7A11

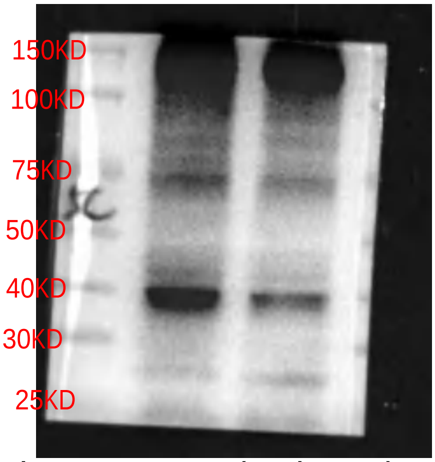

sFigure7I-Tubulin

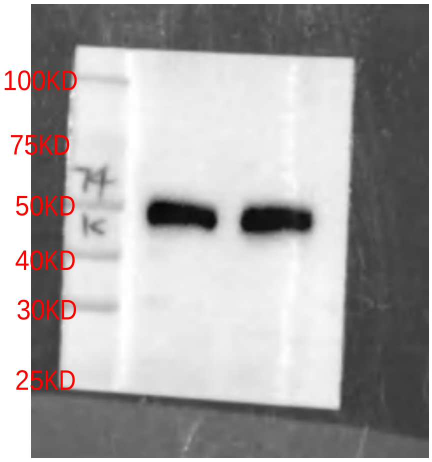

sFigure7I-AMPK(p-Thr172)

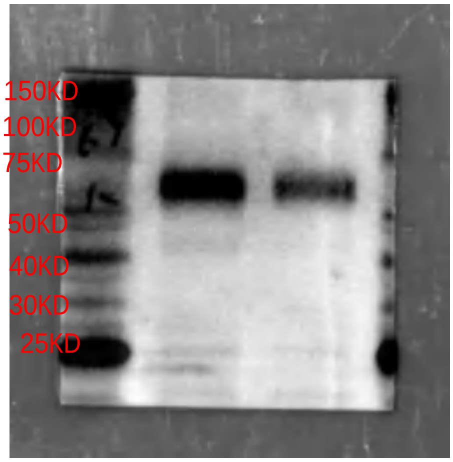

sFigure7I-AMPK

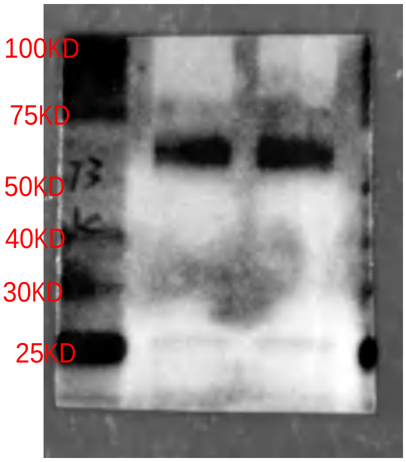

sFigure7I-NRF2

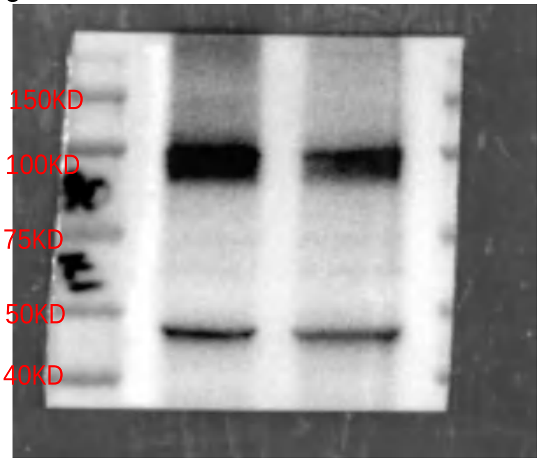

sFigure7I-Lamin B1

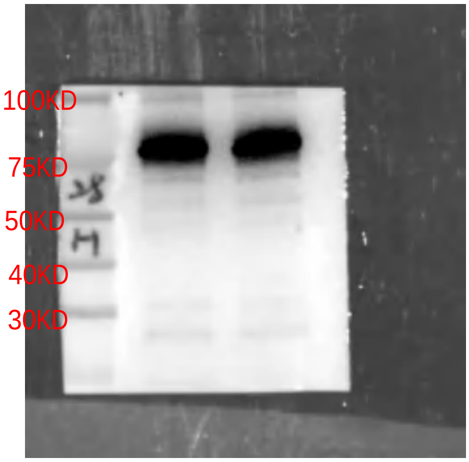

sFigure7J-CAV1

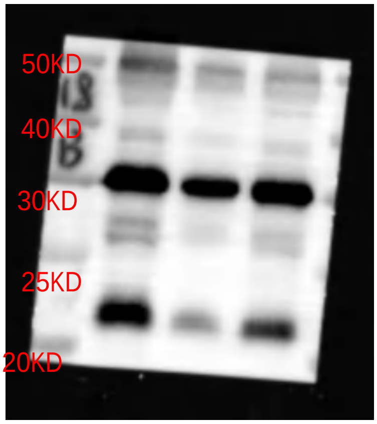

sFigure7J-SLC7A11

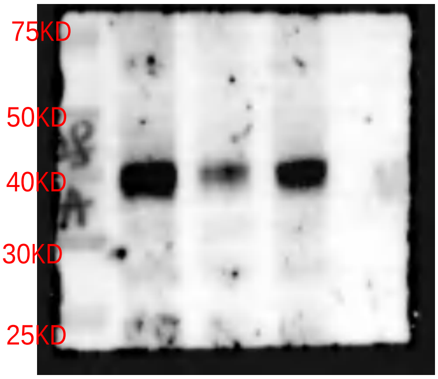

sFigure7J-tubulin

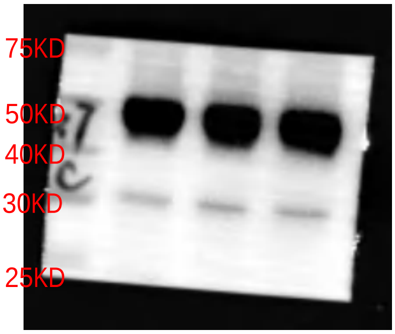

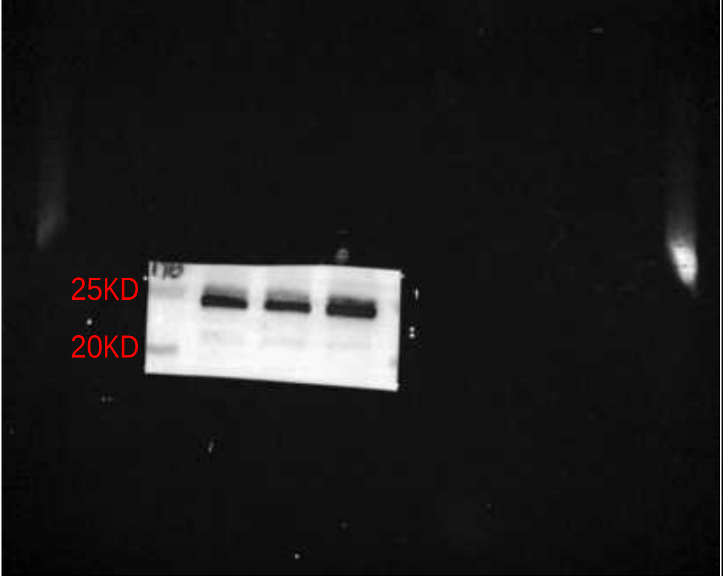

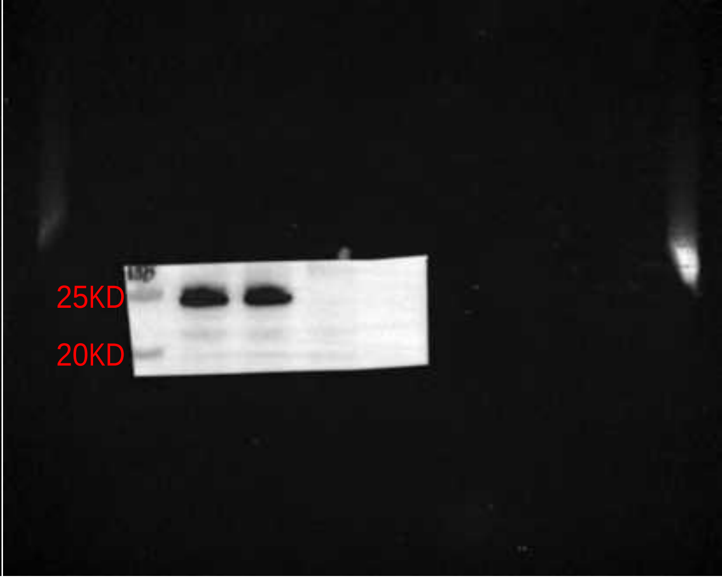

sFigure8A-CAV1

sFigure8B-CAV1

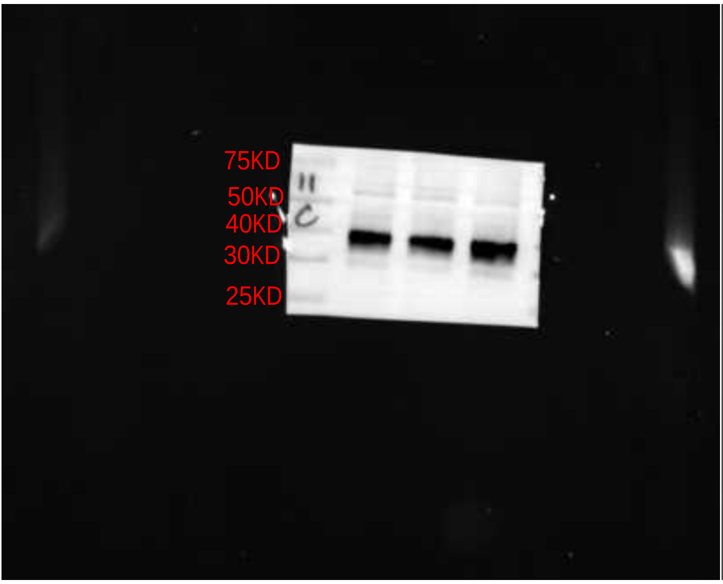

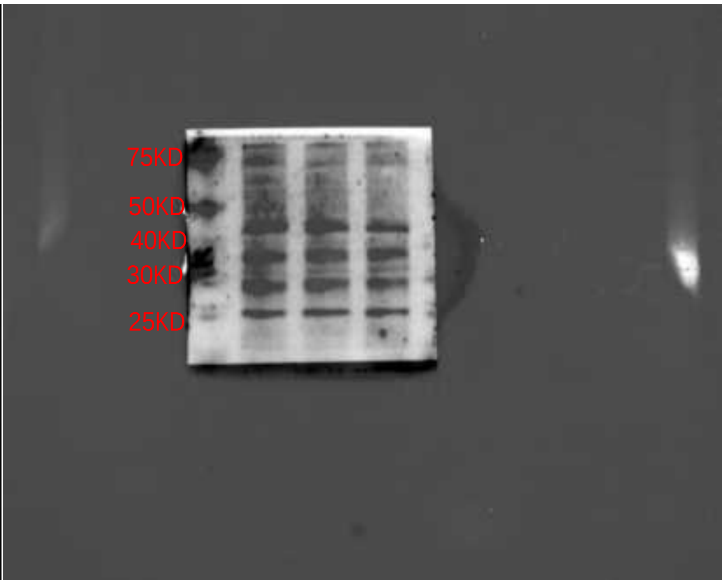

sFigure8A-SLC7A11

sFigure8B-SLC7A11

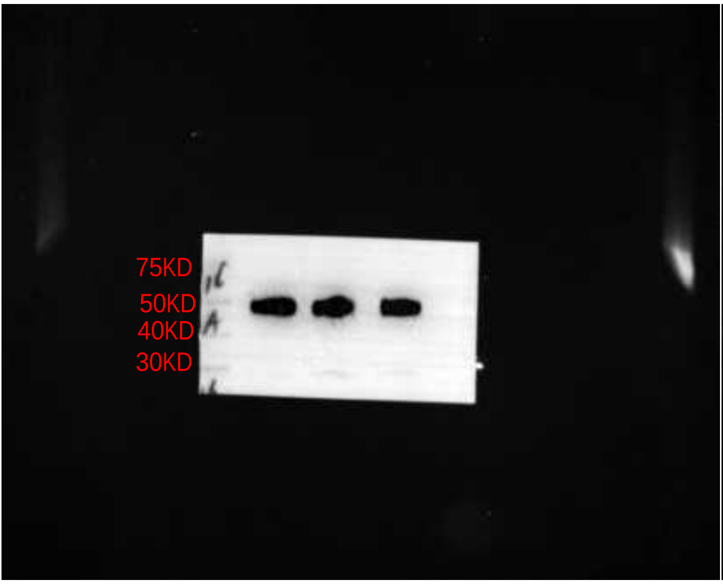

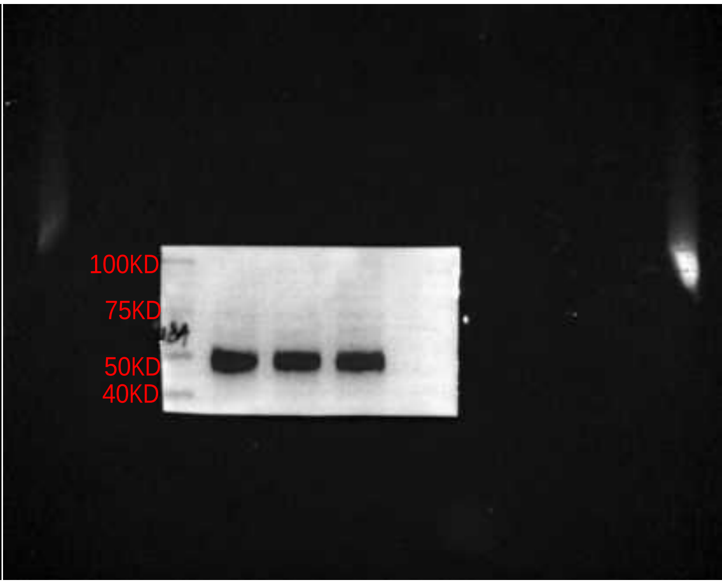

sFigure8A-tubulin

sFigure8B-tubulin

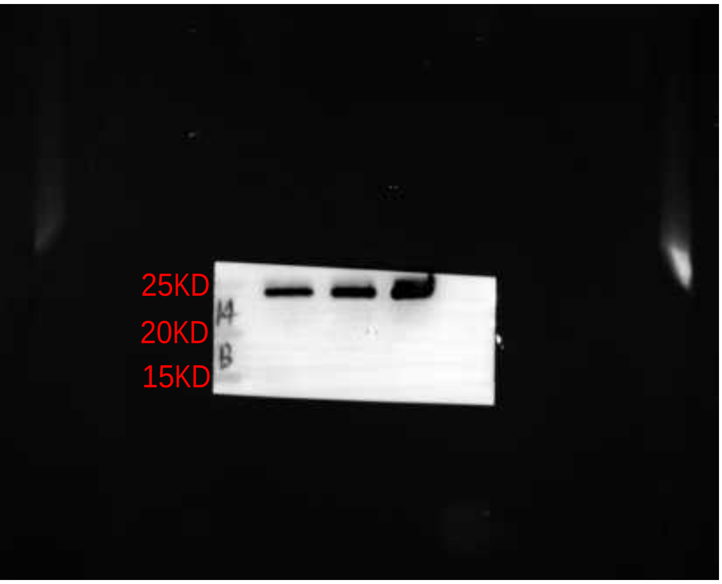

sFigure8C-CAV1

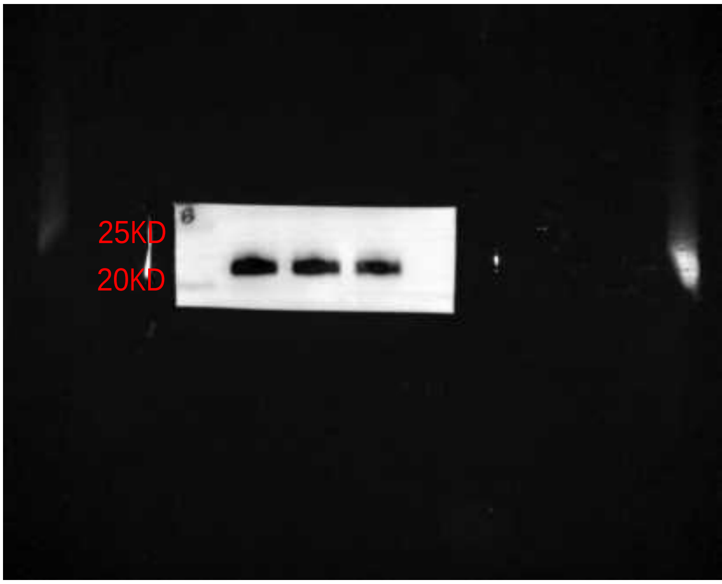

sFigure8D-CAV1

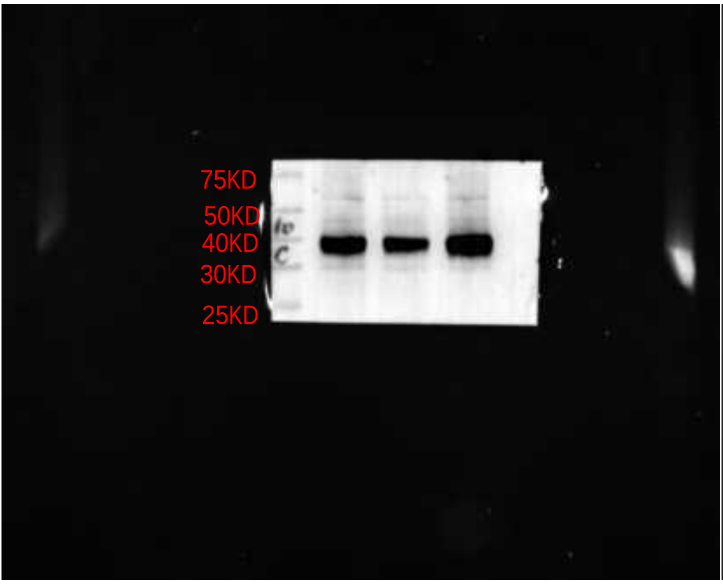

sFigure8C-SLC7A11

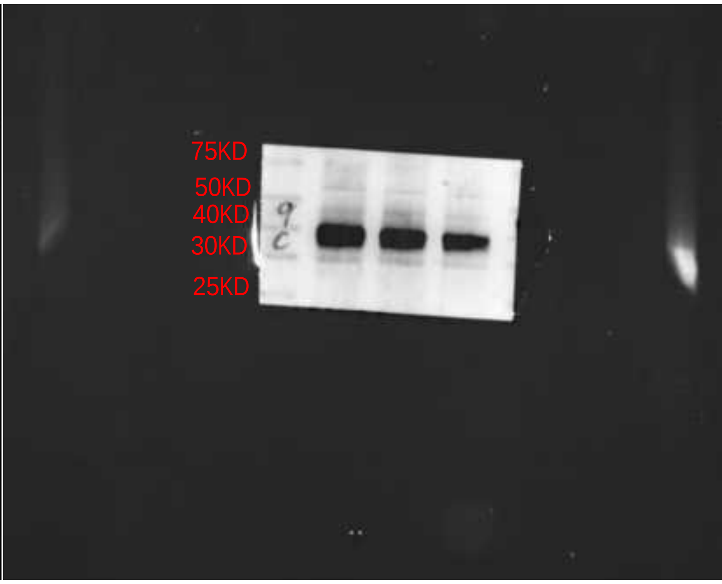

sFigure8D-SLC7A11

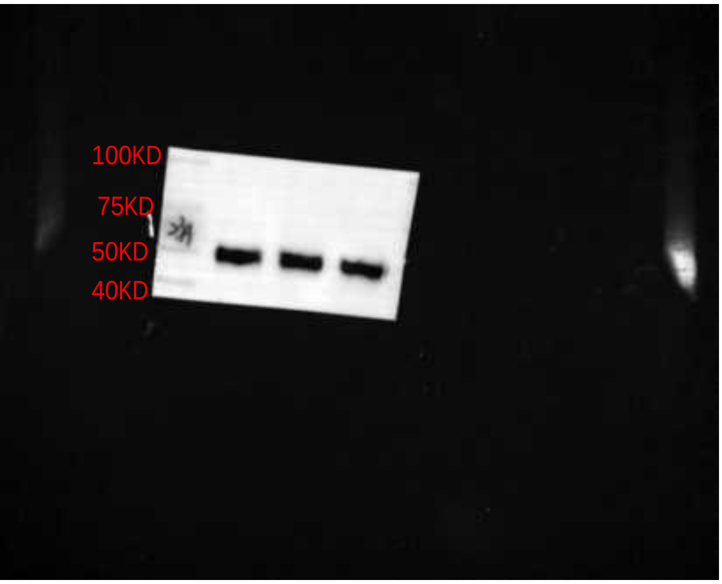

sFigure8C-tubulin

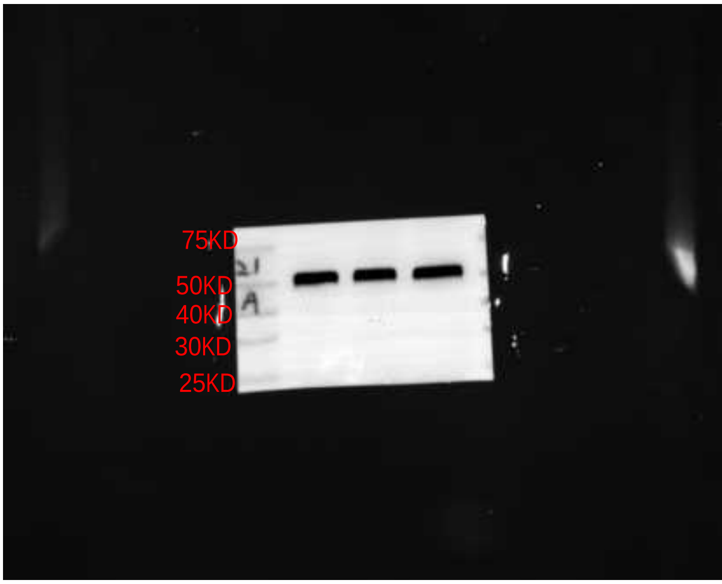

sFigure8D-tubulin
